# Unlocking the Chemical Space for Rechargeable Batteries with a Generative Solvent Design System

**DOI:** 10.1021/acsnano.6c06255

**Published:** 2026-07-16

**Authors:** Zhan-Yun Zhang, Rocío Mercado, Thanh Trung Le, Chao Zhang

**Affiliations:** † Department of Chemistry-Ångström Laboratory, Uppsala University, Lägerhyddsvägen 1, P.O. Box 538, 75121 Uppsala, Sweden; ‡ Wallenberg Initiative Materials Science for Sustainability, 8097 Uppsala University, 75121 Uppsala, Sweden; § Department of Computer Science & Engineering, 11248 Chalmers University of Technology & University of Gothenburg, Chalmersplatsen 1, 412 96 Gothenburg, Sweden

**Keywords:** rechargeable batteries, organic solvent, materials design, machine learning, generative AI

## Abstract

Electrolyte discovery for rechargeable batteries today relies on heuristic trial-and-error or high-throughput screening of existing molecules. Here, we introduce a Generative Solvent Design System (GSDS) that integrates a graph-based deep molecular generator with machine learning (ML) property predictors to design rechargeable battery solvents *de novo*. We enable this by constructing a battery-specific prior data set (Batt-SLM, 115,756 molecules) and fine-tuning a graph-based molecular generator using physics-informed ML surrogates for redox potential, viscosity, melting point, donor number, and dielectric constant. We validated the performance of GSDS on the rediscovery of both fluorinated and phosphorus-containing compounds not seen during training. This allows us to propose application-specific candidates (top 0.2 ‰) for alkali metal batteries—fluorinated diluents and nonfluorinated weakly solvating electrolytes—that pass a posterior verification funnel including property evaluation, synthetic accessibility, candidate prioritization, and literature checks. We conclude that GSDS establishes a tractable solvent design layer of a broader electrolyte-design framework and can be expanded toward salt-aware, interface-informed, and mixture-included optimization for next-generation rechargeable batteries.

## Graphical abstract

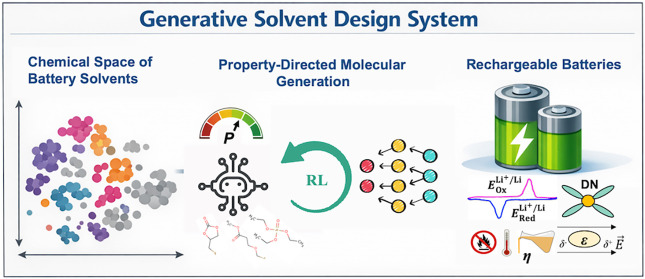

Electrolytes are technologically important for developing sustainable societies through energy storage and conversion devices, such as rechargeable batteries. It was once believed that the electrochemical formation of lithium–graphite intercalation compounds, first observed in the 1950s, was impossible because propylene carbonate (PC), then used as the solvent, decomposes and co-intercalates into graphite at potentials around 0.7 V vs Li^+^/Li, causing exfoliation of the electrode. Yet in the early 1990s, stable lithiation of graphite was finally achieved by replacing PC with ethylene carbonate (EC), which forms a robust solid–electrolyte interphase (SEI) instead of cointercalating. This breakthrough marked an important step toward the modern commercial Li-ion battery. Notably, the absence of a single methyl group in the solvent molecule delayed that advance by nearly 40 years.^1^ The episode underscores how subtle molecular design in electrolytes can dictate the course of battery technology.

For alkali metal rechargeable batteries, the guiding principle of modern electrolyte design is to control the cation (e.g., Li^+^) solvation environment.^2,3^ Highly concentrated electrolytes (HCEs) pioneered this trend:^4,5^ by forcing nearly every solvent molecule to coordinate the cation, they suppress solvent reduction and promote inorganic-rich interphases, albeit at the cost of high viscosity. This concept evolved further into localized highly concentrated electrolytes (LHCEs),^6^ where inert diluents preserve the “anion-rich” local structure of HCEs while lowering viscosity and improving wettability. A complementary strategy emerged with weakly solvating electrolytes (WSEs), which use solvents that interact only weakly with Li^+^ through either low donor number (DN) solvents or sterically shielded donor sites.^7–9^ This allows anions into the first coordination shell, thereby stabilizing Li-metal interfaces and enabling fast ion transport. Finally, fluorinated solvents combine several advantages—lowered donor strength, enhanced oxidative stability, and improved nonflammability—making them a bridge between weakly solvating and chemically stable, high-voltage electrolytes.^10^

Building on these advances, it is also clear that the future of electrolyte design will depend not only on chemical intuition but also on data-driven and computational discovery. Materials modeling provides a powerful framework for predicting electrolyte behavior across the multiple length and time scales that define the reactive liquid environment. First-principles methods—such as density functional theory (DFT) and DFT-based molecular dynamics (MD)—can capture the coupled dynamics of ions, solvents, and interfacial reactions with high fidelity, effectively blurring the boundary between reactive solute and solvent.^11,12^ Yet, the high computational cost of these methods limits their applicability to small systems and short time scales, making it impractical to evaluate properties such as ionic conductivity or to simulate reactive electrode–electrolyte interfaces under realistic operating potentials.^13^ To bridge this gap, surrogate models are needed—approaches that retain much of the predictive power of quantum mechanics while offering orders-of-magnitude faster evaluations.^14–21^ This motivation has led to a cross-fertilization between atomistic machine learning (ML) and generative AI, where models trained on high-fidelity data provide fast and accurate property predictions. Crucially, this enables *inverse design*,^22^ i.e., the automated exploration of electrolyte molecules guided by learned structure–property relationships, which enables researchers to explore beyond high-throughput screening campaigns and known materials.^23–26^

In this work, we developed a Generative Solvent Design System (GSDS) for rechargeable batteries. The work is based on a graph-based deep generative model, of which various instances were trained with a data set, curated herein, of battery solvent-like molecules (Batt-SLM; 115,756 nonduplicated entries), and fine-tuned via property-directed reinforcement learning (RL) for various tasks. The property predictions were made possible by curating available literature data, labeling electronic properties via DFT calculations, and developing a set of physics-inspired ML models as predictors. This enabled us to steer the molecular generator in the GSDS to generate candidates for battery solvents that satisfy multiple constraints, including specific ranges for acceptable redox potentials, viscosities, melting points, donor numbers, and dielectric constants. Through property-directed molecular generation and the posterior verification funnel, we discovered a set of promising solvent molecules in three distinct optimization scenarios for alkali metal batteries: fluorinated diluents, fluorinated WSEs, and nonfluorinated WSEs. Our GSDS framework demonstrates that it is possible to inject chemical intuition via data extracted from the battery literature to learn what properties make sensible electrolytes with a molecular generator and to optimize for battery solvents in an RL setting by coupling the molecular generator with ML-based property predictors.

## Results and Discussion

### Machine Learning Framework of GSDS

The overall workflow of the generative solvent design system (GSDS) developed in this study is illustrated in Figure 1. There are two key components: the molecular generator and property predictors. The molecular generator is a deep neural network implemented in GraphINVENT^27^ and is responsible for generating molecules as 2D molecular graphs. The property predictors, or surrogate models, are a collection of single-task ML models for molecular properties, such as redox potential, viscosity, melting point, donor number, and dielectric constant. Three key ingredients in these ML models are (1) incorporating physical relations in addition to chemical features; (2) linking atomistic ML models from PiNN^28,29^ and descriptor-based ML models; and (3) building on existing experimental and computational data sets for heavy-lifting tasks while also including DFT-labeled data for electronic property prediction. The technical details for the molecular generator and surrogate models can be found in the Methods section.

**Figure 1:**
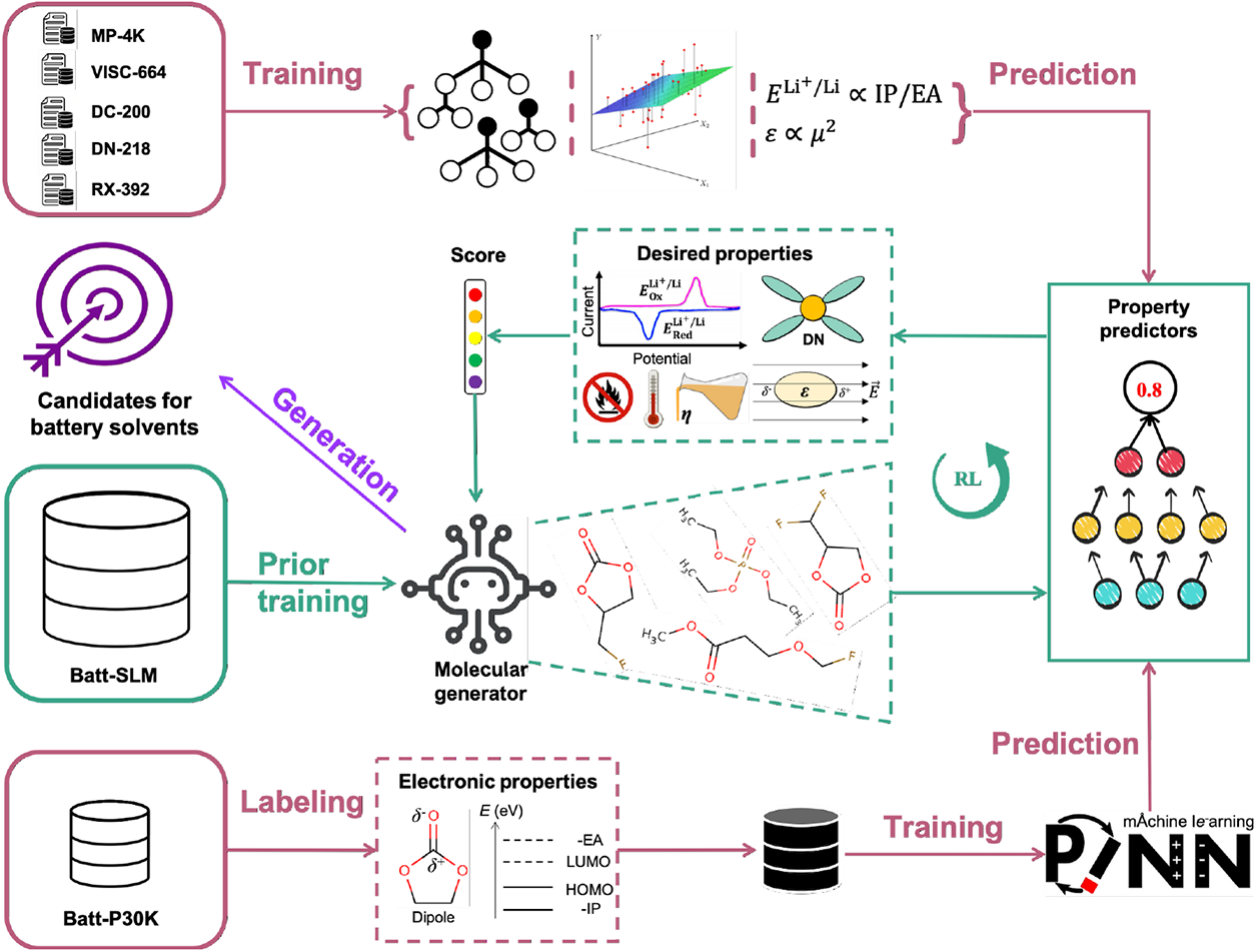
Overall workflow for the Generative Solvent Design System (GSDS). *Prior training*: first, we constructed a chemical dictionary of battery solvent-like molecules (Batt-SLM) without labels and used it to train a graph-based deep molecular generator. *Training the surrogate property predictors*: we then labeled a subset, Batt-P30K, with electronic properties at the DFT level and trained atomistic ML models with PiNN; we collected labeled data sets (MP-4K, VISC-664, DC-200, DN-218, and RX-392) from the literature and built physics-inspired descriptor-based ML models for predicting melting point, viscosity, dielectric constant, donor number, and redox potential. *Property-directed fine-tuning*: next, we fine-tuned the generative prior with application-driven scoring functions using the surrogate property predictors and reinforcement learning (RL). *Final candidate generation*: we generated candidates for battery solvents by using the fine-tuned molecular generators (inference-only).

Before coupling the molecular generator and surrogate models in the RL cycle, they must be trained separately. This requires careful construction and curation of the data sets for each task. For training the prior model in the molecular generator, we constructed a SMILES-based battery-like solvent molecular data set (Batt-SLM) by curating data from the battery literature (this leads to the known battery data set, KBS-409, that we created in this work) as well as incorporating records from the Electrolyte Genome Project,^30^ the MPcules in the Materials Project,^31^ and all PubChem^32^ molecules that are relevant for rechargeable batteries. To train the ML models for property prediction, we have labeled Batt-P30K—a subset of Batt-SLM—with electronic properties (including EA, IP, HOMO/LUMO energies, and molecular dipole moment) using DFT at the hybrid functional level and curated both computational and experimental data for redox potential (RX-392), viscosity (VISC-664), melting point (MP-4K), donor number (DN-218), and dielectric constant (DC-200); note that the integer in each data set name indicates the number of molecules in the data set. The details of data set construction are described in the Methods section.

As shown in Figure 2, Batt-SLM encompasses the chemical space of KBS-409, and the labeled Batt-P30K is indeed a representative subset of Batt-SLM. In addition, the chemical spaces of RX-392, VISC-664, MP-4K, DN-218, and DC-200 also show good overlap with each other and with KBS-409 (see Figure S3a in the Supporting Information). This means the prior model of the molecular generator trained with Batt-SLM has the potential to learn the broad and relevant chemical space for rechargeable batteries and that surrogate property models trained with Batt-P30K and curated literature data should be sufficient to label molecules generated by the molecular generator trained on Batt-SLM.

**Figure 2:**
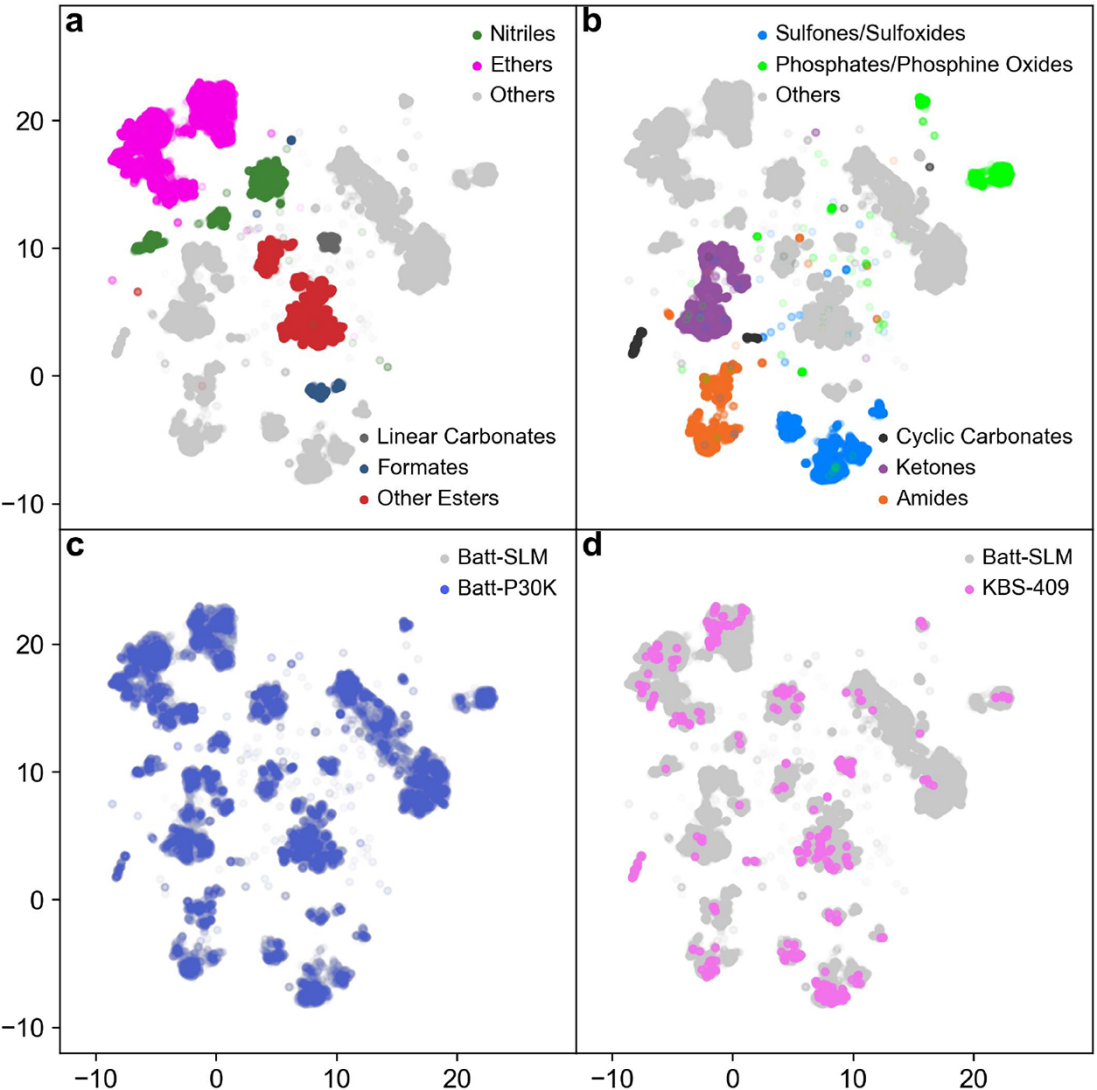
Low-dimensional representation of the chemical space in the GSDS using Uniform Manifold Approximation and Projection (UMAP) and 2048-bit Morgan fingerprints (radius 2). (a, b) Batt-SLM data set, colored according to molecule class; (c) Batt-SLM and Batt-P30K data sets, illustrating significant overlap (Batt-P30K is a subset of Batt-SLM); (d) KBS-409 and Batt-SLM data sets. See the Methods section for the description on the data set naming and construction.

We then developed property predictors for fine-tuning the molecular generators in the GSDS according to the available data. In the high data regime (Batt-P30K), we employed the atomistic ML package PiNN^28,29^ for the following regression tasks: HOMO energy, LUMO energy, EA, IP, and molecular dipole moment prediction. In the low-data regime (VISC-664, MP-4K, DN-218, DC-200, and RX-392), we instead used physics-inspired descriptor-based ML models. We further exploited the coupling between the descriptor-based ML models and the atomistic ML models to predict redox potentials, dielectric constants, and donor numbers (see the Methods section).

The performance of our ML models on the various property prediction tasks is summarized in Figure 3. Atomistic ML models based on PiNet2-P3 show excellent capabilities in capturing the conformational dependence of electronic properties (Figures 3a and S6). Additionally, we find that specific conformers have a significant impact on the molecular dipole moment, where at least three conformers are needed to obtain a proper estimate (Table S3 in the Supporting Information). We therefore consistently use three conformers for predicting electronic properties (*i.e*., IP and EA) for each generated molecule. For liquid electrolytes, Hall et al.^33^ have investigated the redox potentials of 27 commonly used organic solvents in lithium-ion batteries via DFT. As shown in Figure 3b, by combining IP/EA from PiNet2-P3 and the linear correlation (see the Methods section for details), our ML model performs quite well on this oxidation potential benchmark (less well for reduction potentials as compared to the DFT reference but actually more close to experiments, see Figure S7 in the Supporting Information). The same applies to the predictions of the donor number (DN) and the dielectric constant (DC), where the predictions from our models are on par with or better than the state-of-the-art (SOTA) ML models^34^ for these tasks (see Figure 3c,d for the results on DN-218 and DC-200, respectively).

**Figure 3:**
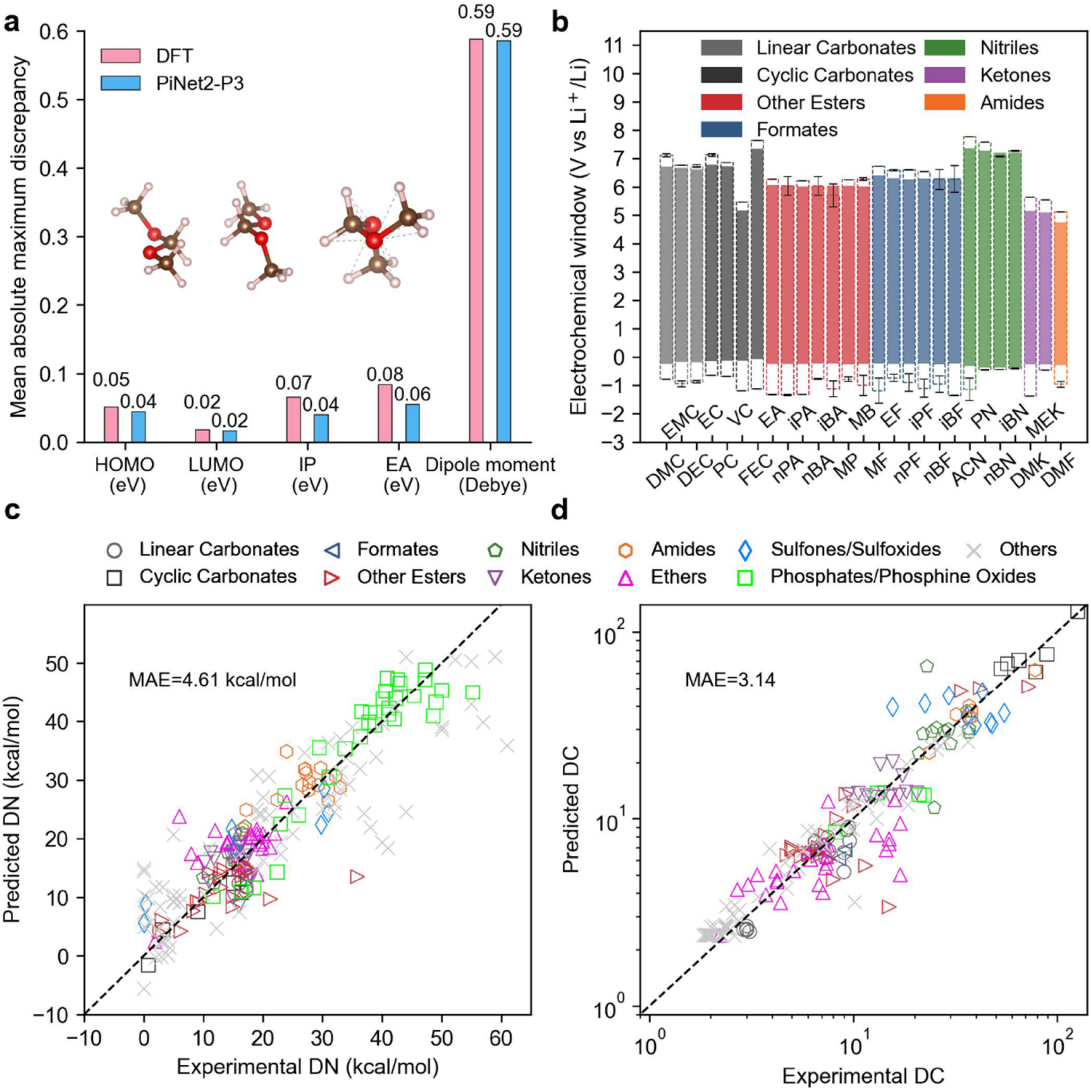
Performance of surrogate property predictors on electronic properties of solvent molecules as well as their redox potentials, donor numbers, and dielectric constants. (a) Mean absolute maximum discrepancies among three conformers for 138 battery solvent-like molecules in the DC-200 data set. PiNet2-P3 is an atomistic ML model (see the Methods section for details). Inset: three conformers of dimethoxymethane which show a large conformation-dependence of dipole moment (>2.0 D). (b) Predicted reduction and oxidation potentials for 27 known battery solvents from Hall et al.^33^ The dashed lines are DFT references, and the error bars are the standard deviation due to conformers. The solid bars are ML predicted results. (c) Parity plot for the experimental and predicted donor numbers (DN) of molecules in the DN-218 data set. (d) Parity plot for the experimental and predicted dielectric constants (DC) of molecules in the DC-200 data set.

### Rediscovery of Fluorinated or Phosphorus-Containing Solvent Molecules

Before steering GSDS toward the discovery of battery solvents with specific property requirements, it is necessary to first demonstrate its validity. For this purpose, we have carried out a rediscovery case study for fluorinated or phosphorus-containing solvent molecules.

We have trained two types of generative priors: one (denoted as prior I) is based on Batt-SLM, and the other (denoted as prior II) is based on a data set of 117,372 molecules; this data set was constructed in a similar way to Batt-SLM but without ever seeing the F/P-holdout data set (see Section 3 in the Supporting Information for details). Then, we have also designed two scoring functions: *S* ^1^(*G*) and *S* ^2^(*G*), where *S* ^2^(*G*) intends to reward the molecular generator to generate F/P-containing molecules, while *S* ^1^(*G*) is the control (see the Methods section for the list of different scoring functions and their components). This leads to four specific combinations of the generative prior and scoring model. As shown in Figure S16, both the average loss and the negative log likelihood (NLL) for taking the correct actions converge after about 800 steps without any signs of overfitting; the model variance is negligible for both metrics.

Now, we are ready to see the results from this rediscovery exercise. As shown in Figure 4a, regardless of the choice of scoring model for fine-tuning, prior I always shows a higher rediscovery rate compared with prior II. This does not come as a surprise because prior I was trained with Batt-SLM, where the F/P-holdout is a part of the data set, which is not the case for prior II. Then, we also found that the combination of prior I + *S* ^2^(*G*) gives the best rediscovery rate. In addition, the case of prior II + *S* ^2^(*G*) shows a rediscovery rate much higher than that of prior I + *S* ^1^(*G*). This suggests that by introducing the Tanimoto similarity measure with respect to the F/P-holdout in *S* ^2^(*G*), GSDS was better steered toward the generation of molecules more likely to contain fluorine or phosphorus atoms. Compared to the baseline, i.e., the fine-tuning steps = 0 in Figure 4a, the rediscovery rate increases by 2.5- to 4-fold with *S* ^2^(*G*) on. It works even better with more complex scoring functions used in the follow-up applications (about a 30-fold increment with the same budget of 20 000 as shown in Figure S18f in the Supporting Information). Therefore, the property-direct fine-tuning is very effective and even more important than the choice of prior. This is also evident in Figure 4c, where the mean maximum Tanimoto similarity of generated molecules to F/P-holdout increases significantly once the corresponding similarity measure is included in the scoring function. Interestingly, we observe that the rediscovery rates for *S* ^2^(*G*) converge after about 25 fine-tuning steps, while the Tanimoto similarity continues to grow. This means the fraction of generated molecules that are not present in the training set should decrease. Indeed, as shown in Figure S17 in the Supporting Information, both the uniqueness and novelty indexes decrease with additional fine-tuning steps. Taking all this into account, it is wise to do early stopping during the fine-tuning process if the goal is to maximize the diversity and novelty of generated molecules matching the desired property constraints.

**Figure 4:**
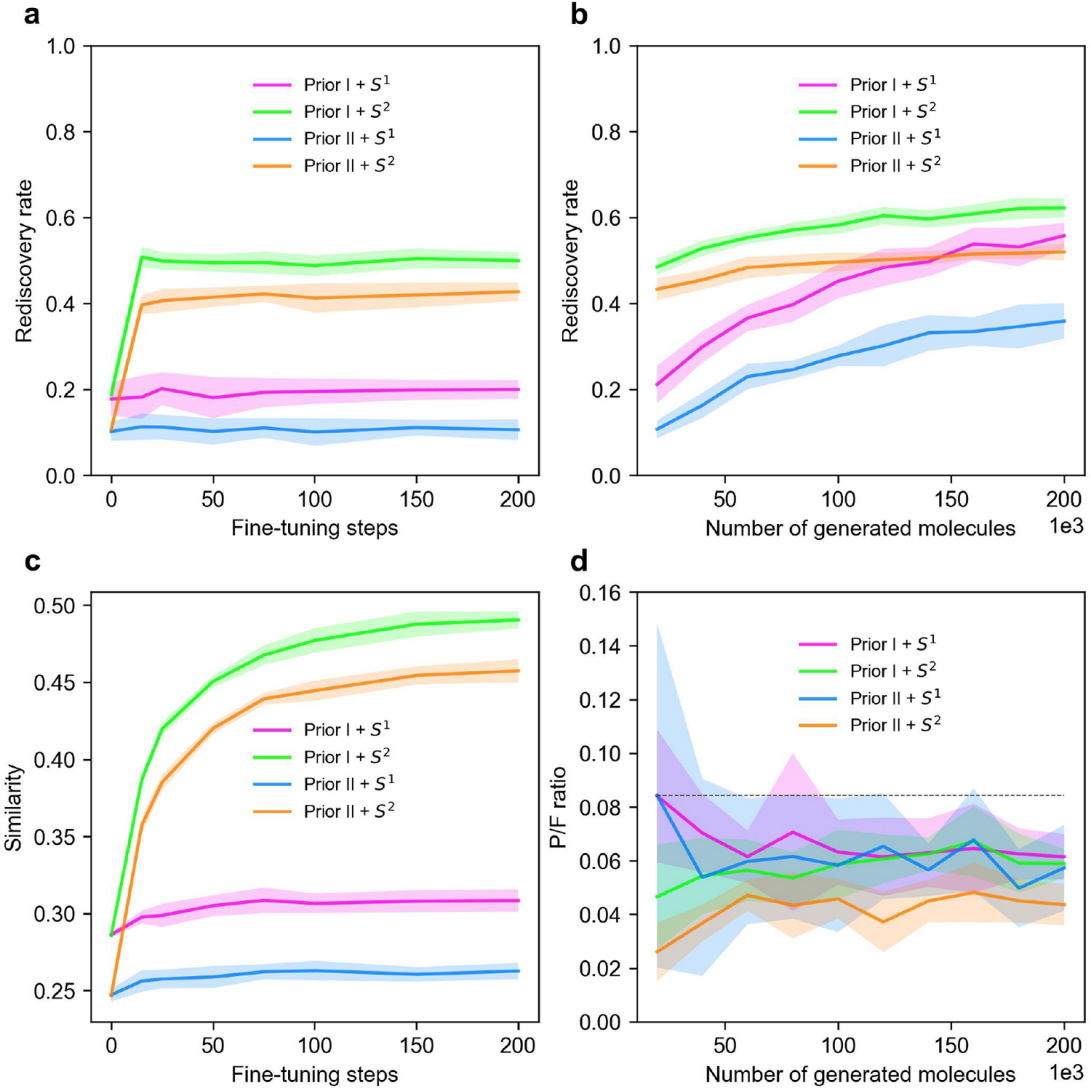
Performance metrics (rediscovery rate, Tanimoto similarity, and P/F ratio, i.e., the number of rediscovered solvents with P relative to those with F) in the rediscovery exercise. (a) Rediscovery rate as a function of the RL (reinforcement learning) steps with different combinations of prior and scoring function (see Main Text). (b) Rediscovery rate as a function of the number of generated molecules. (c) Mean maximum Tanimoto similarity (estimated by 2048-bit Morgan fingerprints with radius 2) as a function of the RL steps. (d) P/F ratio as a function of the number of generated molecules. Dashed line: the average P/F ratio in the F/P-holdout data set. Prior II + *S* ^1^ is the strict blind test, where F/P-holdout was never seen during the construction of the prior and no reward in the RL was given toward generating F/P-containing molecules.

Figure 4a,c was obtained by generating 20,000 molecules from intermediate states of GSDS during fine-tuning. It therefore raises the question whether the rediscovery rate would be different for larger numbers of generated molecules. As shown in Figure 4b, the rediscovery rate increases continuously with the number of sampled molecules, which is expected for a molecular generative model. In the F/P-holdout data set, there are many more fluorinated molecules than those containing phosphorus; in Figure 4d, we explore how the ratio between generated molecules containing phosphorus and those containing fluorine (P versus F) changes with the number of samples. The P/F ratio appears not to be sensitive to the choice of the prior and scoring model, with all models converging after ≈ 100,000 generated molecules.

To summarize, the rediscovery case study for molecules from the F/P-holdout data set shows that the machinery of GSDS is robust and the rediscovery rate is about 0.3 for the strict blind test (Prior II + *S* ^1^) as long as the number of generated molecules is large enough (∼200,000). Then, the fine-tuning process with RL is very effective at increasing the rediscovery rate.

### Discovery of Fluorinated Solvents with Low Donor Number

In the property-directed molecular generation, it is important to have an idea about the design space before fine-tuning the generator via RL. For this purpose, we plot the distribution of reported organic solvent molecules in rechargeable batteries on a donor number (DN)–dielectric constant (DC) quadrant (Figure 5), computed using the surrogate models developed in this work and introduced in the previous section. DN and DC are complementary properties in solvent design as they account for bond polarity and solvent polarity, respectively. As shown in Figure 5a, conventional dilute electrolytes (CDEs) generally occupy the upper-right quadrant; typical examples are ethylene carbonate (EC), acetonitrile (ACN), and dimethyl sulfoxide (DMSO). To improve transport properties, linear carbonates such as ethyl methyl carbonate (EMC) or diethyl carbonate (DEC), in the lower-right quadrant, are often added^35^ to electrolyte formulations. Another example is 1,2-dimethoxyethane (DME), which is usually not preferred as the primary battery solvent due to its very high DN and strong binding ability with Li^+^.^36^ Diluent molecules, used to create localized high-concentration electrolytes (LHCEs), can be found mostly on the left side of the DN–DC quadrant and mainly show DNs < 10 kcal/mol.^37^ In contrast, many of the ether molecules reported as weakly solvating electrolytes (WSEs) can be found on the right side of the quadrant. Nevertheless, it is worth mentioning that among WSEs, a family of fluorinated esters, including methyl 2,3,3,3-tetrafluoropropionate (M4FP),^38^ possess a high DC but a low DN, appearing in the same region as diluent molecules. Figure 5b shows the distribution of F/P-containing battery solvent molecules compared to that of non-F/P-containing ones. It is clear that fluorinated molecules in general show smaller DNs compared to their nonfluorinated counterparts because of a strong electron-withdrawing effect. Based on these observations, we decided to focus on the design of two classes of solvent molecules: (1) fluorinated molecules with a lower DN, which can function as either diluents or WSEs, and (2) nonfluorinated molecules with a lower dielectric constant, which are expected to function as WSEs.

**Figure 5:**
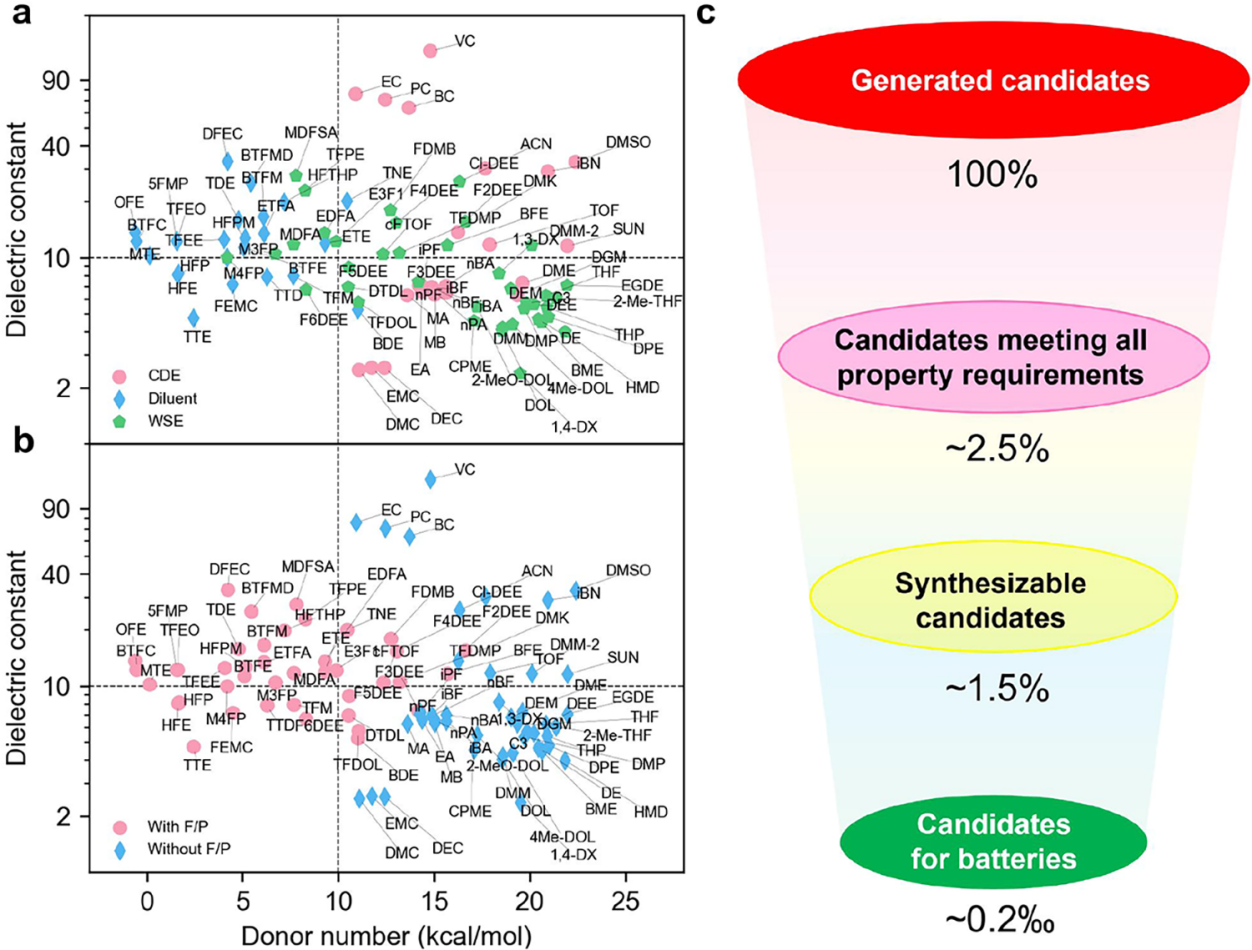
Design roadmap of relevant battery solvents based on the dielectric constant (DC)–donor number (DN) quadrant plots and the verification funnel. (a) DC–DN quadrant colored according to 87 solvent molecules’ primary known functions in rechargeable batteries: conventional dilute electrolyte (CDE), diluent in localized high-concentration electrolytes (Diluent), or weakly solvating electrolyte (WSE). (b) DC–DN quadrant colored according to whether a solvent molecule contains F or P atoms. (c) Illustration of the verification funnel used in the postprocessing of candidate molecules generated by the GSDS (see Section 5 in the SI). The numbers in percent and permille provide an idea about the number of candidate molecules remaining at each stage. DC-DN quadrant plots are for the visualization of the design space. The generated candidates for batteries need to satisfy multiple property constraints (see the Methods section).

Before looking into the results, it is worth walking through the posterior verification funnel used to confirm battery solvent candidates. As shown in Figure 5c, generated candidates from GSDS using the fine-tuned molecular generator are filtered to ensure that they meet all property requirements. Then, synthetic accessibility (SA) score^39^ is used to remove likely unsynthesizable candidates. Here, a threshold of 4.0 was used because most of the solvent molecules in the KBS-409 data set have SA scores below that value (see Figure S22 in the Supporting Information). Finally, we introduced a procedure to make a structurally diverse and novelty-prioritized “promising set” (see Section 5 and Figure S23 in the SI) and manually searched for whether these candidates have been previously reported in the battery literature; molecules that have not been previously reported are considered candidates for use in batteries in this work.

The results of these F/P-containing candidates with donor numbers of less than 10 kcal/mol are displayed in Figure 6. Despite the fact that both fluorinated and phosphorus-containing solvent molecules were encouraged by the scoring function, most of the final generated molecules are fluorinated molecules. Because phosphates/phosphine oxides often have much higher DNs (see Figure 3), which are off-target for the application in this section, this is evidence of the effectiveness of the scoring function and RL-based fine-tuning. Many of the resulting molecules (Figure 6) are fluorinated ethers and esters. In addition, there are promising alternatives, which are fluorinated amides, formates, and ketones. Interestingly, some fluorinated linear carbonates stand out from the other candidates, as this class of molecules is not common for use in either diluents or WSEs. Overall, the diversity shown in Figure 6 highlights the ability of GSDS in exploring the chemical space by leveraging data sets specifically crafted for rechargeable batteries (KBS-409, Batt-SLM, and Batt-P30K) as well as the utility of surrogate models in predicting relevant solvent properties like the DN.

**Figure 6:**
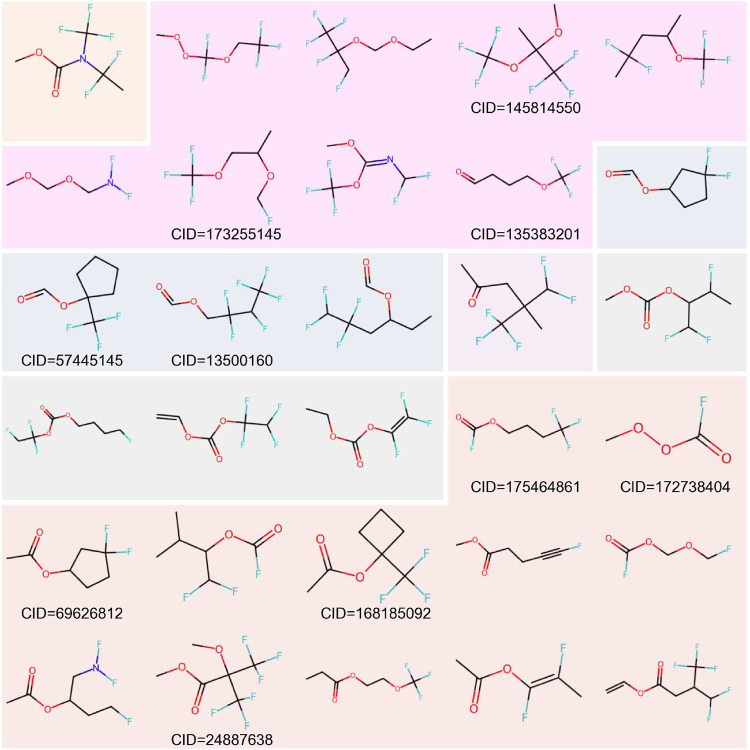
Fluorinated solvents with donor number <10.0 kcal/mol generated by GSDS and passing all checks in the posterior verification funnel. Previously synthesized molecules are illustrated with their PubChem Compound Identifications (CIDs) beneath the molecular structures. None of these candidates have been reported in battery applications at the time of this work. The candidate list with annotated properties can be found in Table S10.

### Discovery of Nonfluorinated Solvents with Low Dielectric Constant

Fluorination of electrolyte solvent molecules has proven to be an effective approach to improve their electrochemical performance while simultaneously alleviating safety concerns for rechargeable batteries.^10^ However, it also raises questions about their long-term environmental impact and recyclability. Therefore, nonfluorinated solvents hold an important position, which has led to a rise in ether-based WSEs and other types of solvents (and salts).^40^ We thus explored GSDS for the design of solvent candidates for batteries that do not contain fluorine atoms yet possess low dielectric constants (ϵ < 10).

Before showing the results, there is another key point worth considering: the role of geometrical accessibility and feasibility in ion solvation. In the DN–DC quadrant, the abilities of different solvents to attenuate the interactions between ions and their intrinsic strengths in donating electrons were mapped out. However, it is known that the multiple intramolecular sites can coordinate Li^+^ and form a chelation structure.^35^ For instance, 1,2-diethoxyethane (DEE) shows a weaker solvating ability as compared to that of DME because of the steric hindrance of the methyl groups.^36^ On the contrary, triethyl orthoformate (TOF) is not ideal as a WSE due to its ability to form multidentate structures with Li^+^.^41^

Taking these two aspects (steric openness and multisite coordination) into account, we developed a chelation propensity index (CPI). Detailed procedures for constructing the CPI are described in the Methods section. As shown in Figure 7a,b, we optimize the CPI through a logistic regression model that includes contributions from both the solvent-accessible surface area (SASA) and the O–O coordination types; this approach works very well to differentiate the solvating abilities of nonfluorinated solvents. Therefore, we have included the CPI in the verification funnel during the postprocessing of candidates generated via GSDS, and only the candidates having CPIs of less than 0.5 are considered potential WSEs in this report.

**Figure 7:**
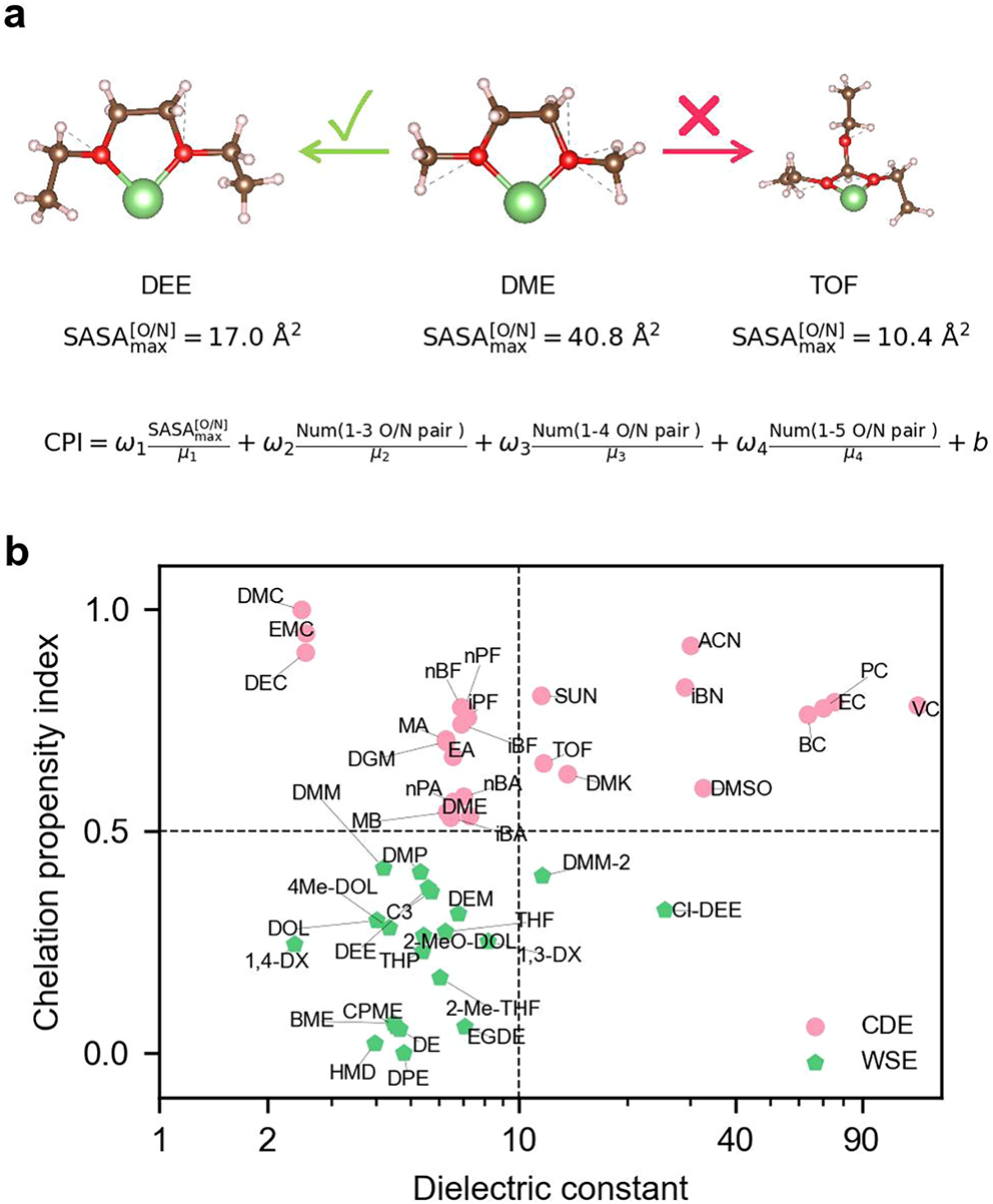
Chelation propensity index (CPI) developed for designing nonfluorinated battery solvents. (a) Examples of dimethoxyethane (DME), 1,2-diethoxyethane (DEE), and triethyl orthoformate (TOF) molecules in terms of their steric openness characterized by SASA and the multisite availability for *n*-member chelates to Li^+^, which leads to the formulation of the CPI (see the Methods section for details). (b) CPI–dielectric constant (DC) quadrant, colored according to 46 solvent molecules’ primary functions in rechargeable batteries: conventional dilute electrolyte (CDE) or weakly solvating electrolyte (WSE).

The resulting solvent candidates for nonfluorinated WSEs that pass the posterior verification funnel are listed in Figure 8. Unsurprisingly, most of these candidates are ether compounds. Nevertheless, a number of these molecules belong to the recently proposed asymmetric ethers, which possess both lithiophilic and lithiophobic groups and show superior electrochemical performance.^42^ Besides ethers, GSDS also identified ester-based alternative solvents. On the one hand, this shows that prior knowledge distilled from the literature has a strong influence on the solvent candidates designed by AI systems, which has also been demonstrated in recent reports.^43^ On the other hand, unexpected candidates were also proposed by GSDS, demonstrating its ability to explore a much broader chemical space for rechargeable battery applications than that known in the literature.

**Figure 8:**
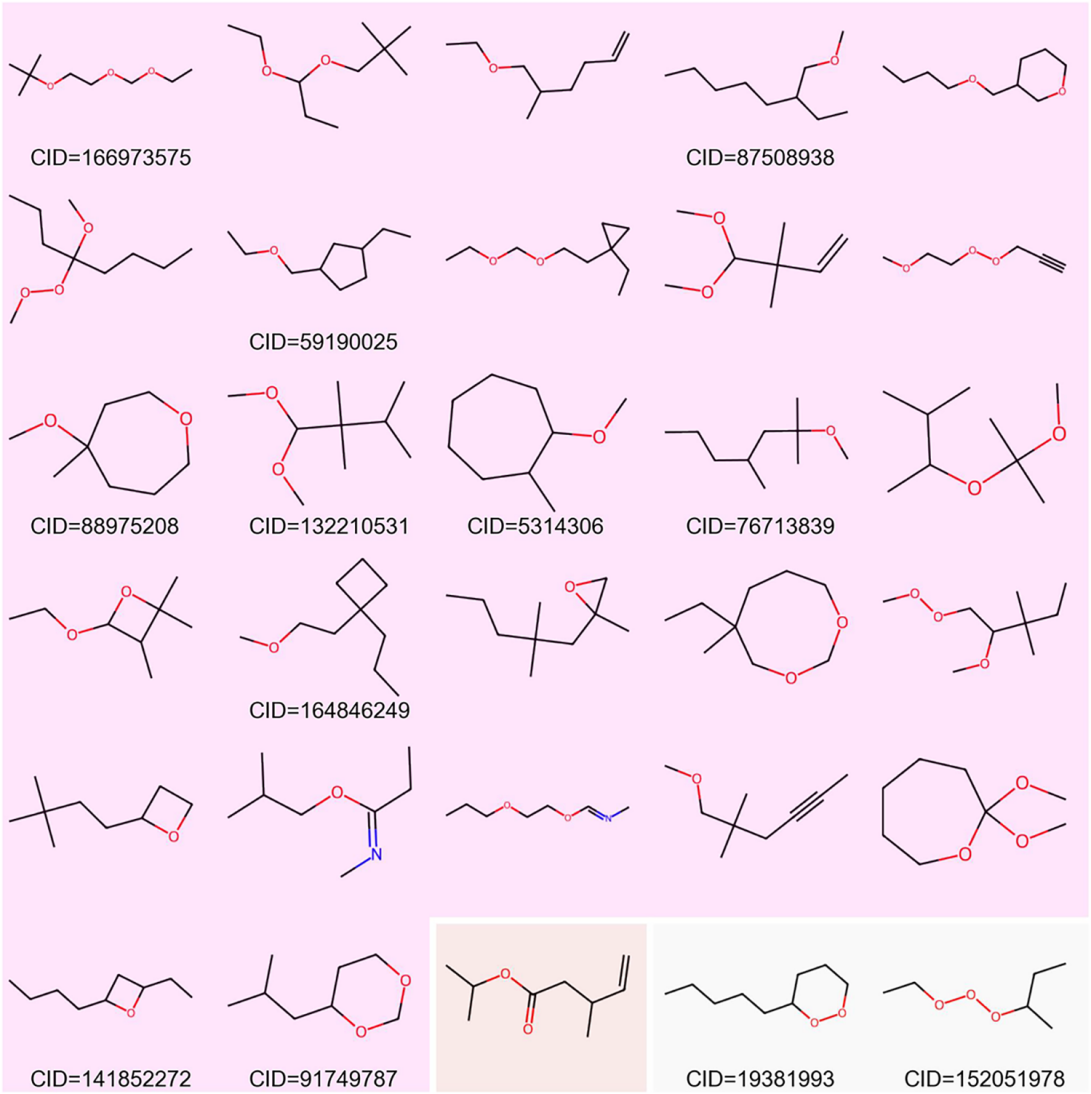
Nonfluorinated solvents with dielectric constant <10.0 generated by the GSDS and passing all checks in the posterior verification funnel. Previously synthesized molecules are illustrated with their PubChem Compound Identifications (CIDs) beneath the molecular structures. None of these candidates have been reported in battery applications at the time of this work. The candidate list with annotated properties can be found in Table S11.

### Outlook

Although GSDS has shown promise for discovering candidate molecules for rechargeable batteries, we emphasize that its current solvent-focused formulation is intended as a tractable first layer of a broader electrolyte-design framework. We see this staged, modular strategy as a practical way to tame the complexity of battery electrolytes, with salt awareness, mixture effects, and interface specificity to be integrated, as outlined below.

Battery electrolytes are intrinsically composite materials; this means that the choice of salts and the chemical composition are equally important when it comes to electrolyte design and discovery. For instance, one key descriptor, donor number, depends significantly on the size and the valence of cations.^44^ Therefore, ML models for property prediction that can take into account the cation information will likely generalize better to a broader range of systems. In addition, the anion becomes crucial when the nature of cations is fixed within a given context, e.g., Li-ion batteries or Na-ion batteries.^45^ In particular, the transport properties are well-known to depend not only on cation–anion interactions but also on anion–anion interactions in concentrated electrolyte solutions.^46,47^ This means that ML models that can predict the concentration dependence and the temperature dependence of transport coefficients will be of great value. These salt-aware property predictors could be plugged into GSDS so as to steer the generation of candidate solvents according to the type of cation, the choice of anion, and the salt concentration.

In contrast to the canonical binary system composed of one type of solvent and one type of salt, most practical battery electrolytes are multicomponent mixtures. At the solvent level, a suitable battery electrolyte solvent should combine several key properties, including sufficient electrochemical stability within the operating voltage window, low viscosity for fast transport, a liquid-state temperature range compatible with operation, appropriate donor ability for cation solvation, and sufficient dielectric constant to support salt dissociation and electrostatic screening. In practice, these requirements are often difficult to satisfy with a single molecule, which is why solvent mixtures are widely used to balance stability, transport, solvation, and salt dissociation.^48–50^ For instance, the fluorinated diluents discovered by GSDS can only be used together with a primary solvent to create LHCEs. With the increase in the number of components and the choice of different mixing ratios, the design space becomes combinatorial. Therefore, high-throughput experimentation^25,51,52^ and molecular simulation^53^ will become indispensable to supply enough data to train models that can tackle this challenge. On the other hand, despite the general expectation of “more is better,” the benefits of high-component mixtures are yet to be demonstrated in rechargeable batteries. In this regard, the nonfluorinated ethers proposed by GSDS do hold significant promise as single-salt-single-solvent electrolytes (4SEs).^8^

Speaking of data, although we have made significant efforts in this work to curate both experimental and computational data from the literature and created data sets such as VISC-664, MP-4K, DN-218, DC-200, and RX-393, the limitation of data size on the prediction accuracy of data-driven surrogate models is real. It would be highly desirable to increase the current experimental data set size by a factor of 100 through large-scale initiatives and long-term collaborative projects. From the computational data point of view, it becomes pressingly urgent to develop methods to compute challenging materials properties such as dielectric constant, viscosity, and ionic conductivity and to generate computational databases for liquids (polymers) and their mixtures (blends). This infrastructure development will have a long-term and significant impact on liquid electrolyte design, similar to what we have witnessed on the crystalline counterparts led by initiatives such as the Materials Project.^54^

While GSDS demonstrates the potential of AI-driven discovery for tailoring solvent properties, its current scope remains confined to the molecular level. The model generates solvent candidates based on bulk descriptors such as dielectric constant, donor number, and viscosity, yet it does not account for the electrode surface environment and the formation of the SEI—factors that ultimately determine battery stability and performance. Capturing these interfacial processes explicitly in materials modeling remains very challenging.^55–57^ Nevertheless, experimental information such as intercalation potentials extracted from cyclic voltammetry could be curated and incorporated into the scoring function, allowing future versions of GSDS to become increasingly interphase-aware, bridging the gap between molecular solvent design and full electrochemical conditions.

The candidates proposed by GSDS in Figures 6 and 8 should be regarded as suitable targets for experimental validation. To support such follow-up studies, we examined the commercial availability of all candidates that satisfy both the property and synthesizability constraints. As summarized in Table S12, 9 candidates from Figures 6 and 8 are commercially available, although their per-gram prices are still relatively high. This reflects a broader challenge in materials discovery: promising compounds may be difficult to adopt experimentally when they are not readily available or affordable, thereby slowing the validation and discovery cycle. Nevertheless, the fact that several commercially available candidates have already been explored in metal battery applications (Table S12 in the Supporting Information) provides encouraging external validation of GSDS.

Finally, when it comes to generative AI, we are optimistic about the promise of emerging techniques such as diffusion models^58,59^ and flow-matching^60,61^ besides the deep graph generative models used in GSDS. From the viewpoint of domain application, the most important ingredient is training the prior, which requires the construction of broad, domain-specific data sets, such as Batt-SLM for rechargeable batteries. We therefore expect future generative models for liquid electrolyte design to benefit greatly from the Batt-SLM data set (https://github.com/Teoroo-CMC/Batt-SLM), extending our ideas further to explore better prior training and fine-tuning strategies for multicomponent electrolyte systems.

## Conclusion

In summary, we constructed Batt-SLM and developed GSDS, a generative workflow that couples a graph-based molecular generator with ML property predictors for rechargeable battery solvent design. The workflow was evaluated by rediscovering fluorinated and phosphorus-containing compounds excluded from prior training and was then applied to solvent generation guided by redox potential, viscosity, melting point, donor number, and dielectric constant. After property filtering, synthetic accessibility assessment, diversity selection, and literature screening, GSDS identified candidate fluorinated diluents and nonfluorinated weakly solvating electrolytes for alkali metal batteries. These results show that literature-derived chemical knowledge and physics-informed surrogate models can guide generative molecular design toward application-relevant electrolyte solvents. The same modular strategy can be extended beyond single solvents to account for salt dependence, solvent mixtures, and interfaces/interphases in rechargeable battery electrolyte design.

## Methods

### Construction of Batt-SLM and Batt-P30K Data Sets

In order to train the prior model used in the molecular generator of GSDS, we needed to construct a molecular data set that simultaneously resembles the reported battery solvent molecules while including a diverse chemical space that would enable the model to extrapolate beyond known battery solvents. We began by collecting 487 different solvent molecules reported in the battery literature (research articles, reviews, and patents),^1,3,10,62–64^ where each entry was manually verified to ensure its relevance. By making histograms of their chemical composition (see Figure S1), we found that most known solvents have <17 heavy atoms, < 2 aromatic rings, and a molecular weight <600 Da. Element-wise, most solvents contain H, C, N, O, F, P, S, and Cl. As expected, they are mostly aprotic; e.g., O–H, N–H, and S–H bonds are nowhere to be found. This helped us to hone in on a core data set of 409 molecules, dubbed Known Battery Solvents (KBS)-409. As molecules containing fluorine/phosphorus atoms are of special interest due to their nonflammability and SEI-forming ability,^65^ we singled out molecules in KBS-409 that contain F/P elements as a holdout data set, called F/P-holdout (174 molecules), for a rediscovery exercise. Further, we found that Tanimoto similarities^66^ (estimated by 2048-bit Morgan fingerprints with radius 2) between the F/P-holdout set and the rest of KBS-409 are >0.20 (see Figure S2a), confirming that they share many similarities.

On the basis of a set of criteria we defined (see Section 1 in the Supporting Information for the exact criteria), we further gathered 1424 molecules from the Electrolyte Genome Project,^30^ 3942 molecules from MPcules,^31^ and 1.2 million molecules from PubChem.^32^ We cleaned up the resulting data set by excluding isotopologues and isomers. As one can see from Figure S2b, the distribution of the number of heavy atoms from PubChem molecules differs significantly from those from other data sets. Therefore, a resampling procedure as described in the Supporting Information was subsequently applied to the PubChem molecules to discourage the count of large molecules and to ensure comparable distributions. This finally leads to the formation of the battery solvent-like molecule data set (denoted as Batt-SLM), which includes 115,756 nonduplicated molecules. The distributions of number of heavy atoms and Uniform Manifold Approximation and Projection (UMAP) features^67^ on KBS-409 and Batt-SLM data sets are visualized in Figures S2c and 2d. The resampling procedure used above did not alter the chemical space of PubChem (Figure S3b) and led to comparable distributions of functional groups between Batt-SLM and PubChem (Figure S3c).

Based on Batt-SLM, we created a subset with 29,519 molecules called Batt-P30K for labeling electronic properties. Batt-P30K includes all 409 molecules from KBS-409, 1424 molecules from the Electrolyte Genome Project, and 3942 molecules from MPcules, as well as 23,744 randomly selected PubChem molecules in Batt-SLM. As shown in Figure 2c, distributions of the Batt-P30K data set and the Batt-SLM data set overlap very well with each other. Initially represented by SMILES strings, we converted the molecules in Batt-P30K to 3D structures according to a two-step procedure. First, 10 conformers were generated per SMILES using the ETKDGv3 method implemented in RDKit,^68,69^ with each structure then optimized using the MMFF94 force field.^70–72^ Next, the conformer structure with the lowest MMFF94 energy was further relaxed using the MACE-OFF23 model through the atomic simulation environment (ASE) package.^73,74^ Finally, electronic structure calculations were carried out at the ωB97X-V/def2-TZVPPD/SMD­(ε = 18.5) level of theory^75–77^ using the ORCA 5.0.4 package.^78^ The distributions of HOMO, LUMO, IP, EA, and dipole moment in the Batt-P30K data set are shown in Figure S5. These data were used later in the prediction of the redox potential as well as the dielectric constant (see Methods Section).

### Compilation of Literature Data Sets on Solvent Properties

Besides the Batt-SLM and the Batt-P30K data sets constructed specially for building the GSDS models, we have also curated computational and experimental data on solvent properties. This allows GSDS to make the best use of previous knowledge.

For the redox potential, which defines the electrochemical stability window of a battery solvent, we extracted 392 neutral organic molecules from the MPcules database^31^ (named RX-392), which is an extension of the Materials Project to molecular properties. After analyzing this RX-392 data set, we found that the EA/IP exhibit almost linear correlations with the corresponding reduction/oxidation free energies (see Figure S4). Therefore, these correlations can serve as levers to estimate the redox potentials once the EA/IP are known. Accordingly, the EA/IP computed for the Batt-P30K data set were done at the ωB97X-V/def2-TZVPPD/SMD­(*ε* = 18.5) level, which is the same as in MPcules.

For the dynamical viscosity, which shows an inverse relationship with the ionic conductivity following Walden’s rule, we gathered 664 experimental viscosity records at room temperature from the literature^62,63,79,80^ and refer to it as the VISC-664 data set. We observe that the viscosity shows a positive correlation with the number of heavy atoms in molecules (Figure S8). Noticeably, only a few records show values of less than 2.0 cP when the number of heavy atoms exceeds 15, which further justifies the choice of 17 heavy atoms as the upper limit for constructing Batt-SLM.

For the melting point, which determines the physical state of solvents under working conditions, Chen and co-workers^24^ compiled a melting-point data set comprising 4,235 molecules with potential electrolyte applications. In this work, we directly adopt this data set and refer to it as MP-4K. The distribution of melting points is shown in Figure S10.

For the donor number, which quantifies the Lewis basicity of a given solvent, we collected 218 experimental records at room temperature from the literature^34,81^ and refer to it as the DN-218 data set. The distribution of donor numbers is shown in Figure S12a.

For the dielectric constant, which gauges the ability of solvent to screen the ionic charge, we curated 200 aprotic samples measured experimentally at room temperature from the literature and public databases^82,83^ and refer to this data set as DC-200. The distribution of dielectric constants is shown in Figure S12b.

### Machine Learning Models for Molecular Generation

We have tailored the GraphINVENT code^27^ as the molecular generator in GSDS. GraphINVENT is composed of a graph-based deep molecular generative model and a RL framework with user-specified scoring functions. To train the prior model in GraphINVENT, 5000 molecules from Batt-SLM were randomly selected as the test set, and the remaining molecules were randomly split into training and validation sets with a ratio of 9:1. The training set molecules were broken into a sequence of actions to “reconstruct” each molecule, starting from an empty graph, using three possible options: add atom, add bond, and terminate graph. During training, a total of 1000 epochs were executed, with an initial learning rate of 1 × 10^–4^, which decreased by a factor of 0.95 every 100 epochs. The batch size of subgraphs was 1000, and the accumulated gradient from 512 batches was utilized to optimize the model parameters. To increase efficiency, we treated hydrogen atoms implicitly and kekulized aromatic bonds. To consider the model variance, we trained three independent prior models using the same split ratio (for training and validation data sets) but different random splits.

After training the prior models, the molecular generator in GSDS was fine-tuned by the RL framework with a memory-aware loss function. This directs the generative policy from the prior policy to an agent policy, which exhibits a greater likelihood for the action sequences that yield molecules with desired properties. A multicomponent scoring function *S*(*G*) = (∏_*i*_^*N*^ *S*_*i*_(*G*))^1/*N*^ was used in the RL framework to integrate all concerned properties, where *G* is a given molecule, *N* is the number of scoring components, and *S*_*i*_(*G*) is the corresponding score from the *i*-th molecular property. The various scoring components *S*_*i*_(*G*) used in this work are summarized in Table 1. The scoring functions *S* ^1^(*G*) and *S* ^2^(*G*) have corresponding components *S*_f,1_(*G*) and *S*_f,2_(*G*) · *S*_s,2_(*G*) for the rediscovery of fluorinated or phosphorus-containing solvent molecules. To design fluorinated solvents with low DN, we have used a scoring model with six components *S*_f,2_(*G*) · *S*_s,2_(*G*) · *S*_v_(*G*) · *S*_mp_(*G*) · *S*_dn_(*G*) · *S*_r_(*G*). For the design of nonfluorinated solvents with low DC, we have used another six-component scoring function *S*_f,3_(*G*) · *S*_s,1_(*G*) · *S*_v_(*G*) · *S*_mp_(*G*) · *S*_dc_(*G*) · *S*_r_(*G*). The temperature parameter β was set to 1.0, 100.0, and 1.0 for *S*_dn_(*G*), *S*_r_(*G*), and *S*_dc_(*G*), respectively. According to the benchmark shown in Figure 3b, the reduction potential threshold was set to 0 V, and the corresponding one for the oxidation potential was set to 5 V. More discussions about the choice of β and the form of the scoring function can be found in Section 4, Figures S18, S19, and S20 in the Supporting Information. Three independent generators were fine-tuned for each scoring function using the same prior model to account for variability in fine-tuning. With three independent prior models, this resulted in a total of nine generators per scoring function.

**Table 1:** Definition of Scoring Components Used to Build the Scoring Models for the Various Tasks Explored in This Work^a^

symbol	value	description
*S*_f,1_	1 if *G* passes all filters; else 0	filters refer to the selection criteria used for constructing Batt-SLM without the Tanimoto similarity measure (see Section 1 in the Supporting Information)
*S*_f,2_	1 if *G* passes all filters; else 0	filters + the requirement for containing F/P elements
*S*_f,3_	0 if *G* passes all filters; else 1	the opposite of *S*_f,2_
*S*_s,1_	maximum Tanimoto similarity of *G* to KBS-409 (excluding F-containing solvents)	
*S*_s,2_	maximum Tanimoto similarity of *G* to the F/P-holdout set	
*S*_v_	1 if η(*G*) ≤ 2.0 cP; else 0	η: viscosity
*S*_mp_	1 if MP (*G*) ≤ 313.15 K; else 0	MP: melting point
*S*_dn_	1/{1 + exp [β(DN(*G*) – 10.0)]}	DN: donor number; β is the temperature parameter in the logistic function
*S*_dc_	1/{1 + exp [β(DC(*G*) – 10.0)]}	DC: dielectric constant
*S*_r_	1/{1 + exp [β(5.0 – *E*_ox_^Li^+^/Li^(*G*))]} if *E*_red_^Li^+^/Li^(*G*) < 0; else 0	*E*_ox_^Li^+^/Li^: oxidation potential; *E*_red_^Li^+^/Li^: reduction potential

a Each component is evaluated per generated molecule (*G*).

The scaling factor for the contribution from the best agent so far to the loss function was set to 0.5. The weight of the contribution from the scoring function to the loss function was set to 20. In total, we used 200 RL fine-tuning steps and evaluated the model every 5 steps. The OneCycleLR learning rate scheduler was used to adjust the learning rate with an initial value of 1 × 10^–5^, a minimal value of 1 × 10^–7^, and a maximal value of 1 × 10^–5^. The batch size during fine-tuning was set to 256. In the design of fluorinated/nonfluorinated solvents, we stopped the fine-tuning process early after 100 steps.

### Machine Learning Models for Property Prediction

The end-to-end PiNet2-P3 architecture implemented in the PiNN code encodes higher-order tensorial features into a graph convolutional neural network and shows an impressive boost in the prediction accuracy with moderate computational overhead.^28^ Hyperparameters used to train PiNet2-P3 models on the Batt-P30K data set are given in Table S1. A total of 3 million gradient descent steps were performed by using the Adam optimizer^84^ with a batch size of 10. The initial learning rate was set to 0.0001, and reduced every 100,000 steps at a decay rate of 0.98 for the HOMO/LUMO and EA/IP models, while the decay steps and decay rate for the molecular dipole moment model were 10,000 and 0.994, respectively. For each property, three independent PiNet2-P3 models were constructed using the same training/validation ratio (9:1) but different random data splits. The mean performance (on the validation data set) of the three final PiNet2-P3 models on HOMO, LUMO, IP, EA, and dipole moment prediction is listed in Table S2.

The oxidation and reduction potentials for a given single-electron transfer reaction can be written as^85^

1 $$E_{\mathrm{ox}}^{{\mathrm{Li}}^{+}/\mathrm{Li}}=\frac{\Delta G_{\mathrm{ox}}}{F}-1.44V$$

2 $$E_{\mathrm{red}}^{{\mathrm{Li}}^{+}/\mathrm{Li}}=-\frac{\Delta G_{\mathrm{red}}}{F}-1.44V$$

where Δ*G*_ox_ and Δ*G*_red_ are the Gibbs free energy changes of the oxidation reaction and the reduction reaction, respectively; *F* is the Faraday constant; 1.44 V is the absolute potential of the Li^+^/Li couple. Here we combined the prediction from the PiNet2-P3 model for EA/IP and the linear correlations between EA/IP and reduction/oxidization free energies found in the MPcules database (see Figure S4). Given that both MPcules and Batt-P30K were constructed at the same level of DFT theory, this strategy provides a very cost-effective way (see Figures 3b and S7) to get access to the redox potential of a given molecule by avoiding computationally expensive calculations for the zero-point energy and solvent reorganization energy, and by utilizing the knowledge from the Materials Project (MPcules).

For the prediction of viscosity using the VISC-664 data set, we built an ensemble classification model to identify whether the viscosity of a specific molecule is ≤ 2.0 cP. The model was composed of three XGBoost-based classifiers, each trained on different random data set splits (also known as bootstrap aggregating or bagging; training:test = 9:1), with the final prediction determined via a hard voting strategy. The XGBoost algorithm was chosen due to its excellent learning capabilities and reduced tendency to overfit on small data sets.^86^ A data point in VISC-664 with viscosity ≤ 2.0 cP was labeled as class 1; otherwise, it was labeled as class 0. The weight-balancing strategy was utilized to mitigate class imbalance, assigning higher weights to underrepresented classes during model training. All the molecular features are listed in Table S4. The hyperparameters were optimized by a grid search method using 5-fold cross-validation on the training set. The performance of the base classifiers and ensemble model is listed in Table S5, and the feature importance is shown in Figure S9. The technical discussions regarding whether or not one should apply the same criteria for constructing Batt-SLM to VISC-664 before classification can also be found in the Supporting Information and Table S6.

For the prediction of melting point on the MP-4K data set, the same approach as for viscosity was employed with a classification decision threshold of 313.15 K. Specifically, samples with melting points ≤ 313.15 K were labeled as class 1, and the remaining samples were labeled as class 0. The performance of the base classifiers and ensemble model is summarized in Table S7, and the feature importance is presented in Figure S11. The technical discussions regarding whether or not one should apply the same criteria for constructing Batt-SLM to MP-4K before classification can also be found in the Supporting Information and Table S8.

The donor number is defined as the reaction enthalpy for the 1:1 adduct reaction between antimony pentachloride (SbCl_5_) and the solvent S at low concentration in the main solvent 1,2-dichloroethane (DCE).^87^

3 $$S\cdot \mathrm{DCE}+\mathrm{DCE}\cdot \left({\mathrm{SbCl}}_{5}\right)⇌S\cdot \left({\mathrm{SbCl}}_{5}\right)+2\cdot \mathrm{DCE}$$

Miranda-Quintana and Smiatek^81^ derived the expression for this reaction energy from the conceptual DFT where only HOMO/LUMO energies are needed (see eq S1 in the Supporting Information). Then, a linear relationship DN (kcal/mol) = −*a*Δ*E*(eV) + *b* was used to fit the Δ*E* to the experimental donor number (see Figure S13a). We found that although this approach works nicely for the demonstrated 15 solvents in the original publication, it performs poorly for the DN-218 data set (see Figure S13b). Therefore, we constructed an ensemble regressor to map molecular features to donor numbers. The ensemble comprised three multilinear regression (MLR)-based learners trained on different random splits of the data set (training:test = 9:1). The base learners yielded MAE and RMSE values of 4.78 ± 0.98 and 6.62 ± 1.51 kcal/mol on the test sets, while the ensemble model achieved 4.61 and 6.34 kcal/mol on the whole data set, respectively. The parity plot between experimental and predicted results for DN-218 and the permutation feature importance are shown in Figures S13c and S13d. The technical discussions regarding whether applying the same criteria for constructing Batt-SLM to filter DN-218 would lead to a better model performance or not for battery-like solvent molecules can also be found in the Supporting Information and Figure S14.

Based on the Kirkwood-Onsager equation, the relationship between molecular dipole moment μ and dielectric constant *ε* can be expressed as^88^

4 $$\frac{4\pi \beta N\mu^{2}g_{K}}{\Omega }=\frac{\left(\varepsilon -1\right)\left(2\varepsilon +1\right)}{\epsilon }$$

where *N* is the number of molecules in a system of volume Ω, β is the inverse temperature, and *g*_K_ is the Kirkwood *g*-factor.^88^ Since the dielectric constant of organic solvent is much larger than one, the right-hand side of the Kirkwood-Onsager equation becomes 2ε + 1. Then, on the left side of the equation, reported g-factors for aprotic solvents are not too different from one.^89^ These two aspects suggest a quadratic relationship between μ and *ε* as a first approximation. Here, we exploited this idea to bridge the atomistic machine learning and the dielectric constant prediction. First, we computed the molecular dipole moment for DC-200 at the same DFT level of theory as used in Batt-P30K. Then, we applied the piecewise regression depending on the value of the dipole moment. For molecules with dipole moments ≤ 4.0 D, a linear relationship was fitted between the squared dipole moments and their corresponding dielectric constants; for molecules with dipole moments >4.0 D, an MLR-based ensemble model was employed to predict the linear coefficient (see details in Section 2.5 of Supporting Information and Figure S15). It is worth noting that the conformers have a significant impact on the molecular dipole moment (Figure 3a and Table S3); therefore, we have used the averaged molecular dipole moment of the three lowest-energy conformers for each molecule in DC-200 when establishing this correlation shown in Figure S15. This conformer averaging procedure was also applied when linking the prediction of molecular dipole moment from PiNet2-P3 and the predicted dielectric constant for any molecule generated by GSDS during the reinforcement learning.

### Chelation Propensity Index (CPI)

The chelation propensity index (CPI) has been developed in this work to estimate the relative ability for a molecule to form a chelation complexes with cations. For battery organic solvents, oxygen and nitrogen atoms play the most significant roles in chelation due to their high nucleophilicity. Therefore, our definition of CPI is based on the steric characteristics of O/N atoms: (i) the maximal solvent-accessible surface area (SASA) of the O/N atoms and (ii) the involvement of 4-, 5-, or 6-membered rings in chelation. Taking the above two points into consideration, four features were adopted as shown below:

5 $$\mathrm{CPI}=\omega_{1}\frac{{\mathrm{SASA}}_{\max}^{\left[O/N\right]}}{\mu_{1}}+\omega_{2}\frac{\mathrm{Num}\left(1-3O/N_{\mathrm{pair}}\right)}{\mu_{2}}+\omega_{3}\frac{\mathrm{Num}\left(1-4O/N_{\mathrm{pair}}\right)}{\mu_{3}}+\omega_{4}\frac{\mathrm{Num}\left(1-5O/N_{\mathrm{pair}}\right)}{\mu_{4}}+b$$

where μ is the mean of feature values and ω is the linear coefficient.

To establish the CPI, a set of 46 known nonfluorinated battery solvents, along with their reported functions in batteries, were collected from the literature. The best parameters in CPI were obtained by logistic regression using the sequential least-squares programming (SLSQP) algorithm implemented in the SciPy package,^90,91^ with non-negativity constraints imposed on ω. To generate the features, 50 conformers were generated using the ETKDGv3 method implemented in RDKit^68,69^ for each solvent, and all conformers were subsequently optimized using the MMFF94 force field.^70–72^ Then, the four aforementioned features were extracted for all conformers and averaged using Boltzmann weighting based on their MMFF94 potential energies. For the labels, those 46 solvent molecules were assigned a value of 1 for conventional dilute electrolytes (CDE) 1 and a value of 0 for others (i.e., weakly solvating electrolytes). To improve model performance, a margin penalty weighted by 30 was incorporated into the logistic loss function to encourage larger margins between classes.^92^ These led to the optimized values for ω_1_, ω_2_, ω_3_, ω_4_, and *b* as 0.355, 0.110, 0.044, 0.006, and −0.012, respectively. The effectiveness of CPI was validated using random splits and an external test set, as summarized in Table S9 and the accompanying discussion in the Supporting Information. These results provide initial evidence for the transferability of the CPI among nonfluorinated battery solvents, although broader external validation, particularly with more recently reported WSEs, will be needed for a further assessment.

## Supplementary Material



## Notes

Construction of the prior training data set Batt-SLM; construction of property predictors for battery solvents; fine-tuning prior models in the rediscovery of the F/P-holdout; optimization of the scoring functions in reinforcement learning; construction of the posterior verification funnel; and feasible candidates for experimental validation (PDF)
