# Supplementary material for: Unlocking the Chemical Space for Rechargeable Batteries with a Generative Solvent Design System
Source: ACS Nano. 2026 Jul 16;20(29):20714–29. doi: 10.1021/acsnano.6c06255 (PMC13422007; doi:10.1021/acsnano.6c06255)
Supplement: Supplementary file 1 [file nn6c06255_si_001.pdf]

Supporting Information

Unlocking the Chemical Space for Rechargeable Batteries with a Generative Solvent Design System

Zhan-Yun Zhang,[†,‡] Rocío Mercado,[¶] Thanh Trung Le,[†,‡] and Chao Zhang*,[†,‡]

†*Department of Chemistry-Ångström Laboratory, Uppsala University, Lägerhyddsvägen 1, P. O. Box 538, 75121 Uppsala, Sweden*

‡*Wallenberg Initiative Materials Science for Sustainability, Uppsala University, 75121 Uppsala, Sweden*

¶*Department of Computer Science & Engineering, Chalmers University of Technology & University of Gothenburg, Sweden*

E-mail: chao.zhang@kemi.uu.se

Contents

1 Construction of the prior training dataset Batt-SLM **S3**

2 Construction of property predictors for battery solvents **S5**

2.1 ML models for electronic properties (HOMO/LUMO, EA/IP and molecular dipole moment) and redox potentials S6

2.2 ML models for viscosity S11

2.3 ML models for melting point S15

2.4 ML models for donor number S18

2.5 ML models for dielectric constant S22

3 Fine-tuning prior models in the rediscovery of the F/P-holdout **S24**

4 Optimization of the scoring functions in reinforcement learning **S26**

5 Construction of the posterior verification funnel **S33**

6 Feasible candidates for experimental validation **S45**

References **S47**

1 Construction of the prior training dataset Batt-SLM

The final selection criteria used to collect molecules in Batt-SLM are as follows:

- Only including H, C, N, O, F, P, S, and Cl elements
- $2 <$ Num(non-H atoms) < 17, MW < 600 Da, Num(arom. ring) < 2
- Charge neutral and non-radical molecules
- No isotopic or isomeric SMILES
- Containing C, and at least one O or N atom.
- Aprotic, i.e., without O-H, N-H, and S-H bonds
- Mininum Tanimoto similarity to F/P-holdout ≥ 0.20

To maintain a similar distribution of number of heavy atoms on PubChem[1] molecules to that of KBS-409, the following resampling procedure was performed. First, all molecules with less than or equal to 9 heavy atoms were kept. Second, the count of molecules with $9 + M$ heavy atoms ($1 \leq M \leq 6$) were reduced to 1.5 times of the count of molecules with $9 - M$ heavy atoms. Third, the count of molecules with 16 heavy atoms were reduced to 1.5 times of the count of molecules with 3 heavy atoms. The molecules with high Tanimoto similarities,[2] determined using 2048-bit Morgan fingerprints[3] and radius 2 to molecules in F/P-holdout (174 molecules), were selected.

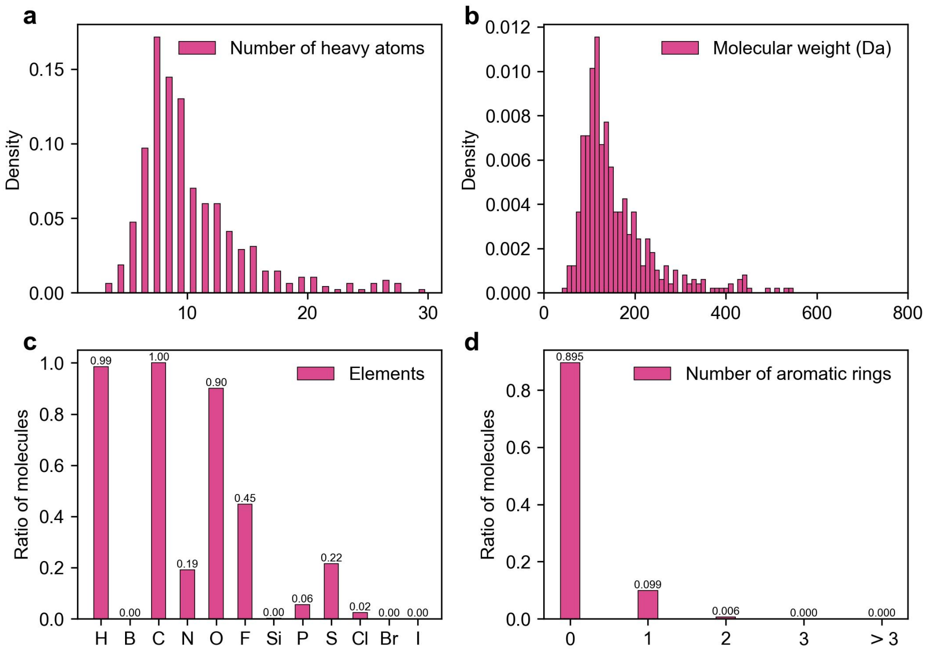

Figure S1: Distribution of number of heavy atoms, molecular weight, elements, and number of aromatic rings for 487 collected known battery solvents. **a** Number of heavy atoms; **b** Molecular weight; **c** Elements; **d** Number of aromatic rings.

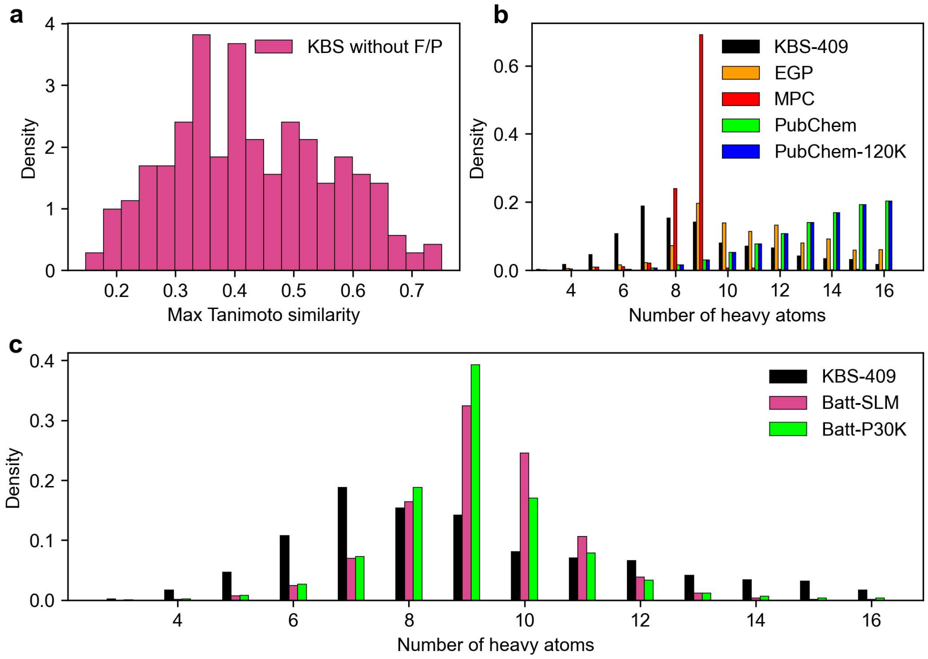

Figure S2: Distribution of Tanimoto similarity and number of heavy atoms for molecules in datasets. **a** Tanimoto similarities of collected known battery solvents (KBS) without F/P atoms to those in F/P-holdout; **b** Number of heavy atoms of molecules in KBS-409, Electrolyte Genome Project (EGP), MPcules (MPC), PubChem, and PubChem-120K; **c** Number of heavy atoms of molecules in the KBS-409, Batt-SLM, and Batt-P30K.

2 Construction of property predictors for battery solvents

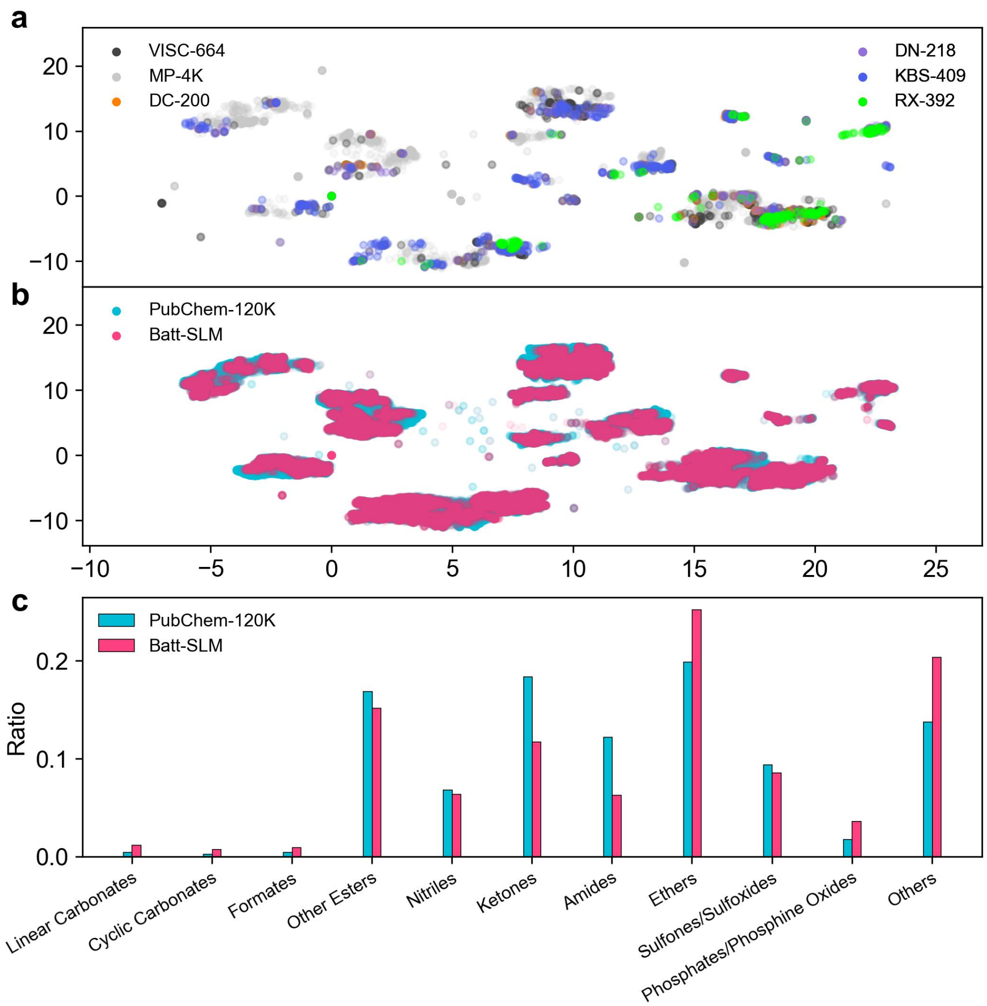

Figure S3: Chemical spaces on 2D features reduced by the UMAP algorithm from 2048-bit Morgan fingerprint and functional group distributions. **a** Chemical spaces of the KBS-409, RX-392, VISC-664, MP-4K, DN-218, and DC-200 datasets; **b** The comparison of the chemical spaces between PubChem-120K and Batt-SLM; **c** Ratios of molecules containing specific functional groups in PubChem-120K and Batt-SLM. PubChem-120K is a control dataset, where 119,995 molecules were uniformly sampled in terms of the number of heavy atoms from PubChem dataset shown in Figure S2b.

2.1 ML models for electronic properties (HOMO/LUMO, EA/IP and molecular dipole moment) and redox potentials

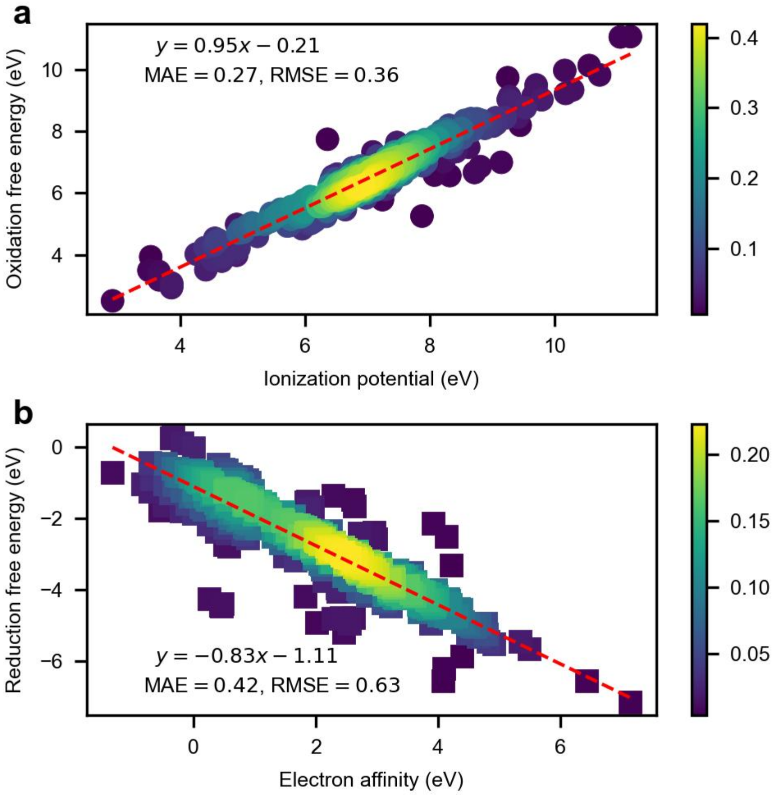

Figure S4: Linear relationships between EA/IP and redox free energies in the RX-392 dataset. **a** Oxidation free energy *vs* IP; **b** Reduction free energy *vs* EA. (colorbar: kernel-density estimate value)

Table S1: Hyperparameters of PiNet2-P3 models on the Batt-P30K dataset for HOMO, LUMO, EA, IP and molecular dipole moment.

Layer	Architecture	Parameter	Value
PI	[64] × 15	R_c	4.5 Å
II	[64,64,64,64]	GC blocks	5
PP	[64,64,64,64]	n_{basis}	15
Output	[64,1]		

Due to the conformer-dependent characteristics of electronic properties, we further investigate the influence of conformers on the calculated and predicted molecular properties. First, 138 battery solvent-like molecules were filtered out from the DC-200 dataset using the

Table S2: Validation performance of PiNet2-P3 models (average of 3 models) in terms of MAE.

model	HOMO (meV)	LUMO (meV)	EA (meV)	IP (meV)	Dipole moment (Debye)
PiNet2-P3	76.55 ± 1.74	53.62 ± 3.36	139.67 ± 6.46	81.72 ± 4.36	0.062 ± 0.002

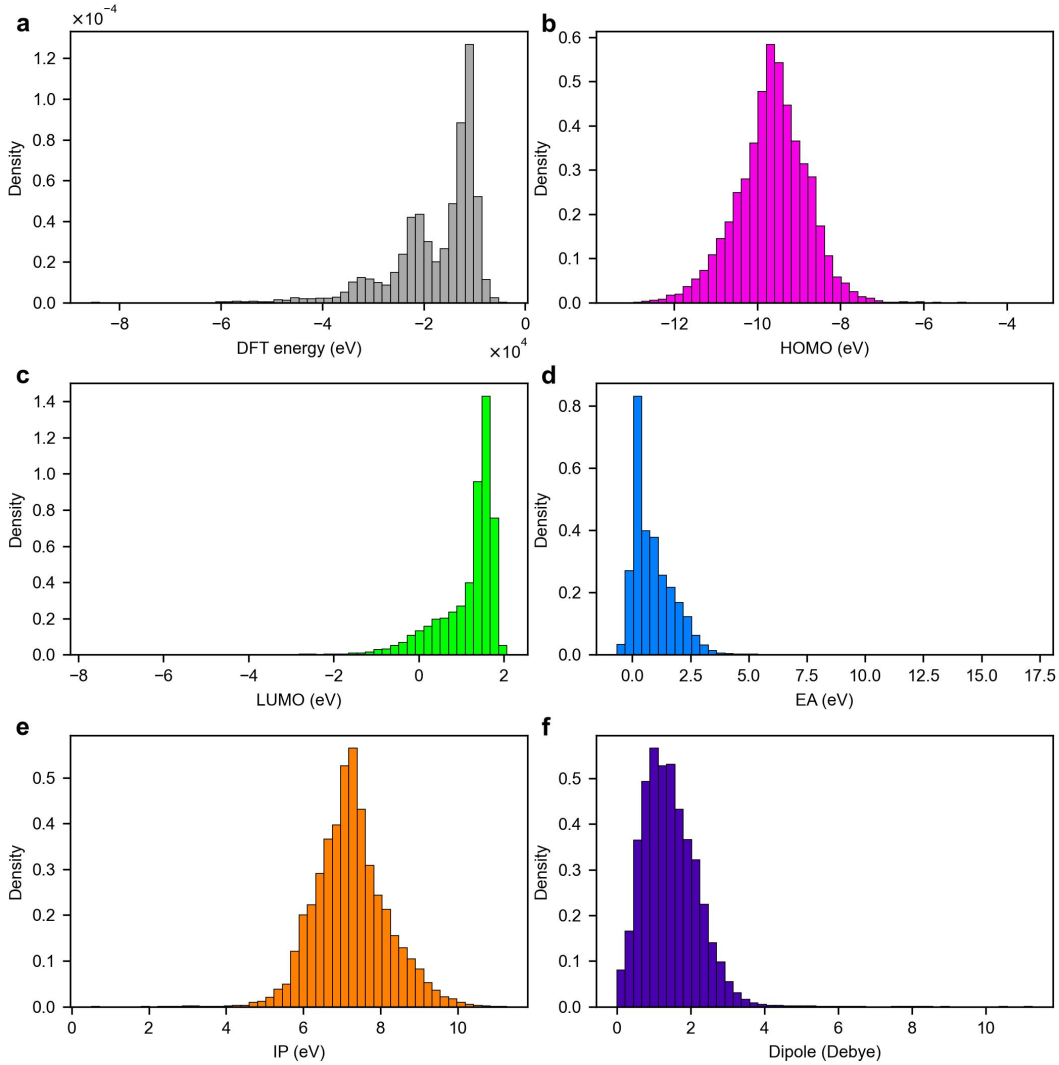

Figure S5: Distribution of calculated molecular properties in the Batt-P30K dataset. **a** DFT energy; **b** HOMO; **c** LUMO; **d** EA; **e** IP; **f** Dipole moment.

filters applied on Batt-SLM. Second, 4 different conformers of each molecule were generated, and their geometry structures were relaxed at the MMFF94 level.[4–6] Third, all conformers were further optimized by the MACE-OFF23 model.[7] Fourth, the five electronic properties of all conformers were computed at the target DFT level and predicted by corresponding PiNet2-P3 models.[8] The averaged maximum variations of the properties among conformers is shown in Table S3. As we can see, the maximum discrepancies of HOMO/LUMO and EA/IP are smaller than 0.1 eV, whereas the corresponding values of molecular dipole moment is larger than 0.5 Debye. It suggests that a minimal of three conformers are needed to obtain accurate estimations for the molecular dipole moments. The performance of PiNet2-P3 model over 3 conformers are illustrated in Fig. S6b-f through a parity plot with the DFT-calculated results. Interestingly, the prediction MAEs on HOMO/LUMO and EA/IP are smaller than 0.07 eV, and the MAE on dipole moment is even smaller than 0.01 Debye. It confirms that the PiNet2-P3 models are capable of making reliable predictions for different conformers, despite only energy-optimized conformers involved in the Batt-P30K dataset. In this context, three conformers were utilized in the workflow, and the final prediction represents the average predicted outcomes from three PiNet2-P3 models over these conformers.

Table S3: Averaged maximum variations among different number of conformers across 138 battery solvent-like molecules in the DC-200 dataset.

method	Number of conformers	HOMO (eV)	LUMO (eV)	EA (eV)	IP (eV)	Dipole moment (Debye)
DFT	3	0.051	0.018	0.084	0.065	0.588
	4	0.062	0.024	0.096	0.091	0.697
PiNet2-P3	3	0.044	0.016	0.055	0.040	0.586
	4	0.057	0.020	0.071	0.052	0.695

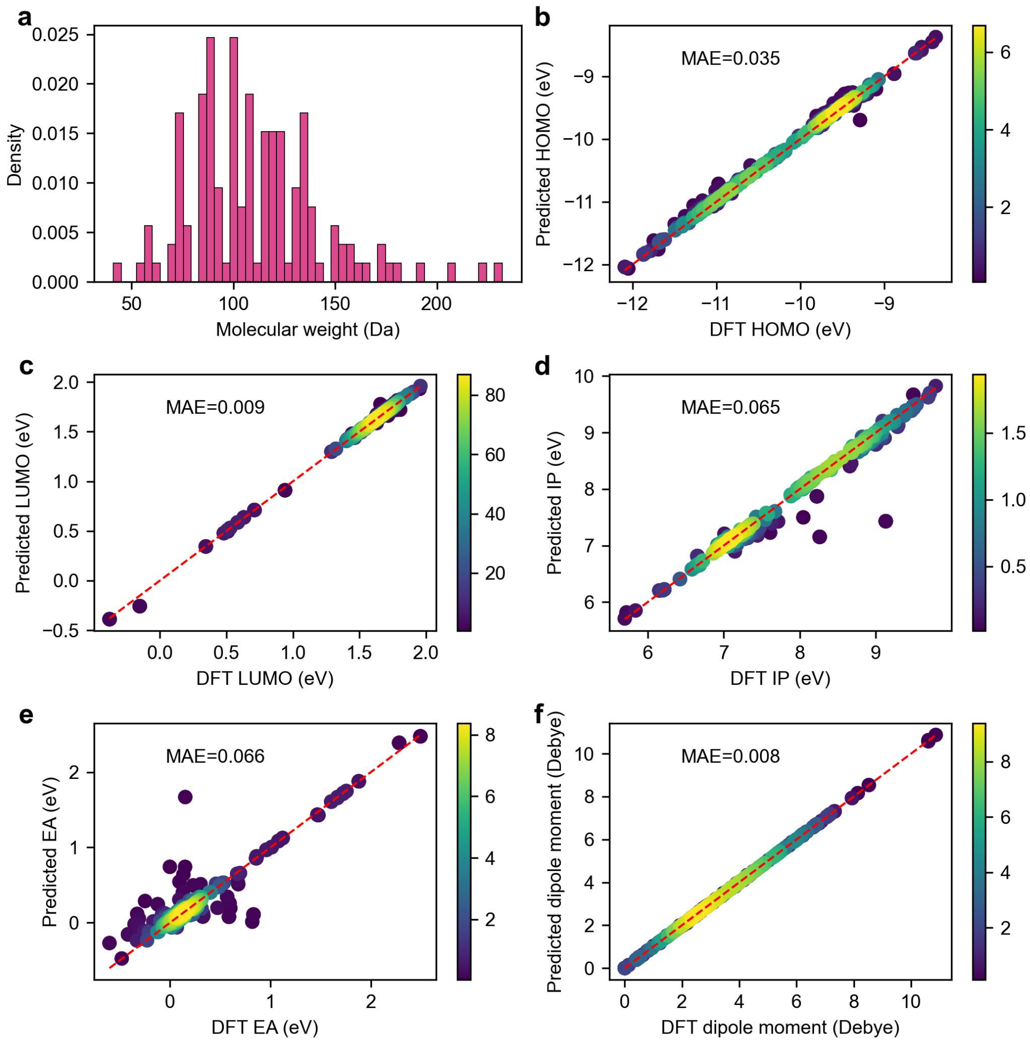

Figure S6: Performance of PiNet2-P3 predictors on the conformers of 138 battery solvent-like molecules in the DC-200 dataset. **a** The distribution of molecular weights; **b-f** Parity plots of DFT calculated properties *vs* PiNet2-P3 predicted results. (colorbar: kernel-density estimate value)

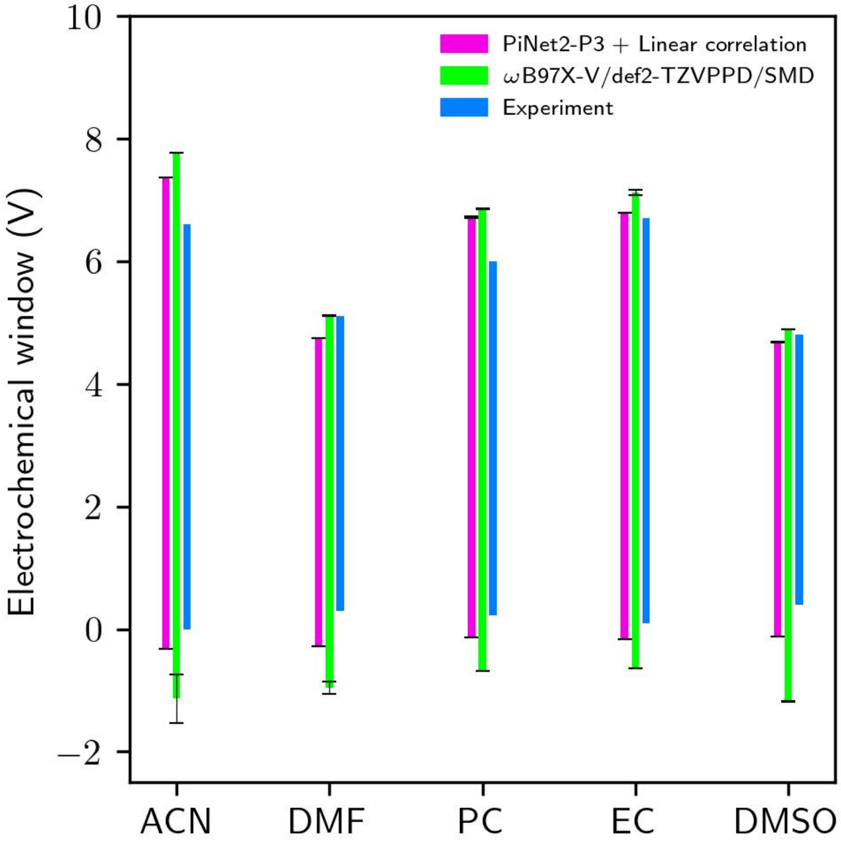

Figure S7: Comparison of the ML predicted, DFT calculated and experimental redox potentials of 5 common battery solvents in Ref.[9] The error bars represent the standard deviations across three conformers.

2.2 ML models for viscosity

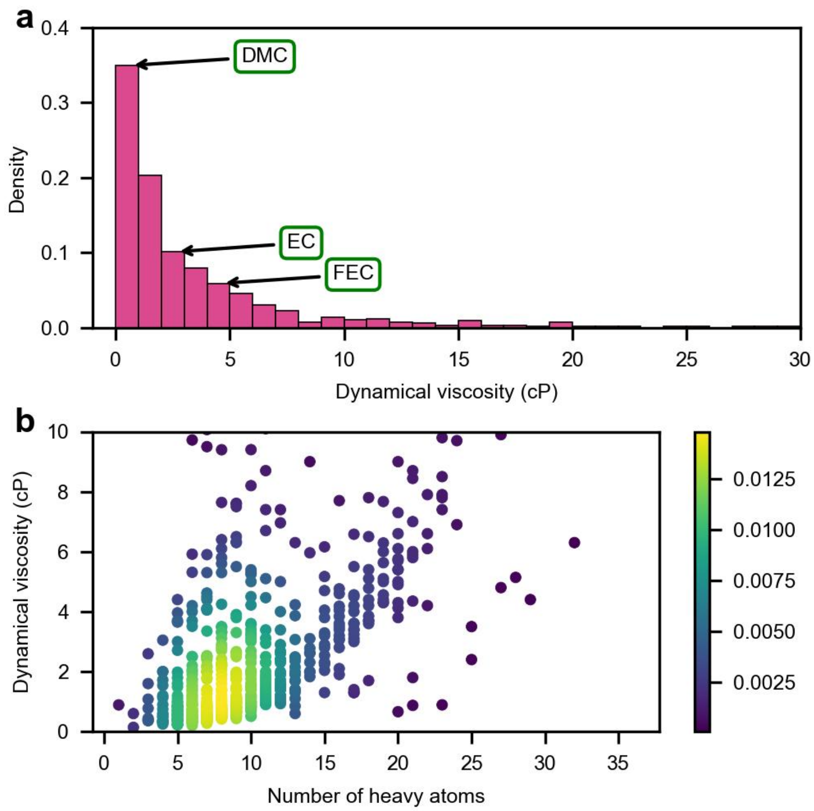

Figure S8: Distribution of experimental dynamical viscosity and number of heavy atoms for molecules in the VISC-664 dataset. **a** Experimental dynamical viscosity; **b** Dynamical viscosity *vs* number of heavy atoms. (colorbar: kernel-density estimate value)

Given the macroscopic nature of viscosity and the scarcity of experimental records, predicting exact viscosity values in the VISC-664 dataset is challenging. Fortunately, our primary objective is to get candidates with low viscosity rather than predicting the true values. Hence, we constructed an ensemble model with hard voting to classify whether the viscosity of a molecule is less than or equal to that of EC (2.0 cP). Three XGBoost base classifiers were trained on different training sets. The hyperparameters, including max depth, number of trees, learning rate, subsample ratio, and colsample_bytree were optimized via the grid search with 5-fold cross-validation strategy. The input features were summarized in Table S4. The performance of the ensemble model (denoted as XGB-VISC-664) is listed in Table S5, and the importance of the top-10 most important features is shown in Fig. S9.

Table S4: Molecular features for models of viscosity, melting point, donor number, and dielectric constant

**feature**	**description**	**feature**	**description**
MolWt	molecular weights	NumValenceElectrons	Number of valence electrons
Num(lone pair)	total number of lone pair	Num(Atom)	total number of atoms
Num(Non-H)	number of heavy atoms	Num(aroAtom)	number of aromatic atoms
AroAtom%	ratio of aromatic atoms	Num(C)	number of carbon atoms
C%	ratio of carbon atoms	Num(N)	number of nitrogen atoms
N%	ratio of nitrogen atoms	Num(O)	number of oxygen atoms
O%	ratio of oxygen atoms	Num(F)	number of fluorine atoms
F%	ratio of fluorine atoms	Num(S)	number of sulfur atoms
S%	ratio of sulfur atoms	Num(P)	number of phosphorus atoms
P%	ratio of phosphorus atoms	Num(X)	number of halogen atoms
X%	ratio of halogen atoms	Num([N/O])	number of nitrogen and oxygen atoms
[N/O]%	ratio of nitrogen and oxygen atoms	NumHeteroatoms	Number of heteroatoms
q_{min}(N)	minimum Gasteiger nitrogen charge	q_{ave}(N)	averaged Gasteiger nitrogen charge
q_{max}(N)	maximum Gasteiger nitrogen charge	q_{sum}(N)	sum of Gasteiger nitrogen charges
q_{min}(O)	minimum Gasteiger oxygen charge	q_{ave}(O)	averaged Gasteiger oxygen charge
q_{max}(O)	maximum Gasteiger oxygen charge	q_{sum}(O)	sum of Gasteiger oxygen charges
q_{min}([N/O])	minimum Gasteiger nitrogen and oxygen charge	q_{ave}([N/O])	averaged Gasteiger nitrogen and oxygen charge
q_{max}([N/O])	maximum Gasteiger nitrogen and oxygen charge	q_{sum}([N/O])	sum of Gasteiger nitrogen and oxygen charges
Num(C-O)	number of C-O bonds	(C-O)%	ratio of C-O bonds
Num(C=O)	number of C=O bonds	(C=O)%	ratio of C=O bonds
Num([C-O/C=O])%	number of C-O and C=O bonds	[C-O/C=O]%	ratio of C-O and C=O bonds
Num(N-O)	number of N-O bonds	Num(N-H)	number of N-H bonds
(N-H)%	ratio of N-H bonds	Num(O-H)	number of O-H bonds
(O-H)%	ratio of O-H bonds	Num([N-H/O-H])	number of N-H and O-H bonds
[N-H/O-H]%	ratio of N-H and O-H bonds	NumHAcceptors	number of hydrogen bond acceptors
NumHDonors	number of hydrogen bond donors	NumAmideBonds	Number of amide bonds
NumRotatableBonds	number of rotatable bonds	NumAliphaticRings	Number of aliphatic rings
NumAromaticRings	number of aromatic rings	NumHeterocycles	Number of heterocycles
NumAliphaticCarbocycles	number of aliphatic carbocycles	NumAliphaticHeterocycles	Number of aliphatic heterocycles
NumAromaticCarbocycles	number of aromatic carbocycles	NumAromaticHeterocycles	Number of aromatic heterocycles
Delta E	reaction energy in DN		

Table S5: Performance of the base classifiers and the ensemble XGB-VISC-664 model.

**model**	**dataset**	**class**	**precision**	**recall**	**F1-score**
base classifiers	3 training sets	0	0.99±0.02	0.99±0.02	0.99±0.02
		1	0.99±0.01	0.99±0.01	0.99±0.01
	3 test sets	0	0.93±0.01	0.90±0.03	0.91±0.02
		1	0.92±0.02	0.95±0.01	0.94±0.01
ensemble model	whole VISC-664 dataset	0	0.99	0.99	0.99
		1	0.99	0.99	0.99
	279 battery	0	0.99	0.97	0.98
	solvent-like molecules	1	0.99	0.99	0.99
	61 battery	0	0.85	0.58	0.69
	solvent-like external molecules	1	0.83	0.95	0.89

We also noticed that only 279 molecules in the VISC-664 dataset are battery solvent-like, i.e., successfully passed through the filters applied on Batt-SLM. It raise another concern that the ensemble model build on the whole VISC-664 dataset may be biased to the molecules differ from battery solvents. Hence, a new ensemble model (denoted as XGB-VISC-279) was built based on these 279 molecules following the same procedure. As we can see from Table S6, the precision, recall, and F1-score of base classifiers on the test sets are considerably lower than those observed on the training set, indicating a significant overfitting problem. The performance on the positive samples (i.e., class 1) from the 279 molecules shows no significant improvement when compared to the performance of XGB-VISC-664 model. To further evaluate the robustness of models, we collected 61 room-temperature viscosity records of battery-like molecules from Ref.[10] (19 negative samples and 42 positive samples), which are not included in the VISC-664 dataset and were used as an external dataset. As shown in Table S5, the external test precision and recall of the XGB-VISC-664 model for positive samples are 0.83 and 0.95, respectively, indicating that most positive samples can be successfully identified. Compared with the XGB-VISC-279 model (see Table S6), the XGB-VISC-664 model exhibits slightly better generalizability. Take these into consideration, the XGB-VISC-664 model was selected as the final predictor.

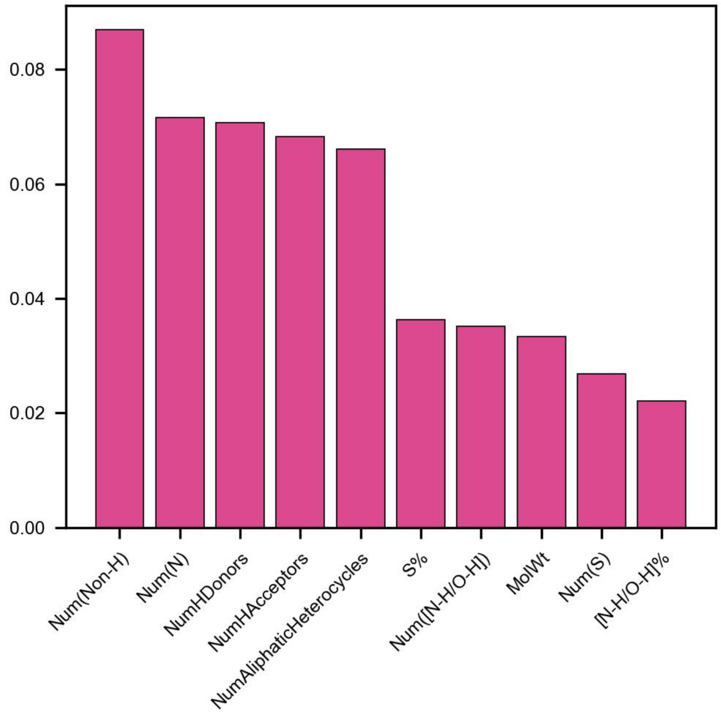

Figure S9: Importance of the top-10 features of the XGB-VISC-664 ensemble model for viscosity.

Table S6: Performance of the base classifiers and the ensemble XGB-VISC-279 model.

**model**	**dataset**	**class**	**precision**	**recall**	**F1-score**
base classifiers	3 training sets	0	0.98±0.01	0.99±0.01	0.99±0.01
		1	0.99±0.01	0.99±0.01	0.99±0.01
	3 test sets	0	0.69±0.02	0.76±0.18	0.72±0.09
		1	0.92±0.06	0.89±0.02	0.90±0.02
ensemble model	279 battery	0	0.97	0.99	0.98
	solvent-like molecules	1	0.99	0.99	0.99
	61 battery	0	0.69	0.47	0.56
	solvent-like external molecules	1	0.79	0.90	0.84

2.3 ML models for melting point

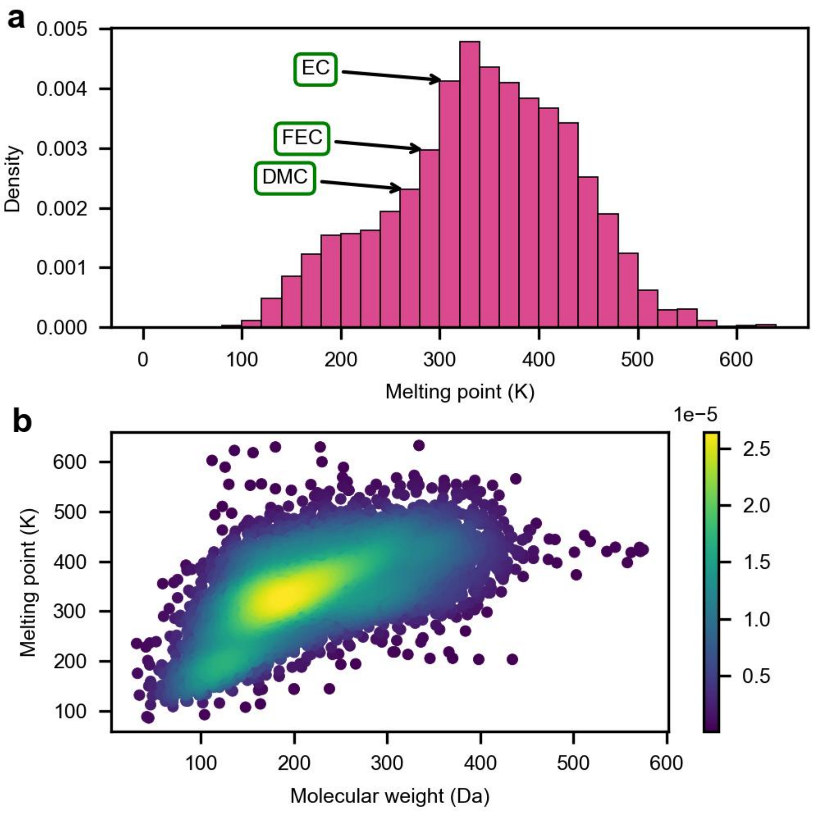

Figure S10: Distribution of experimental melting points and molecular weights for molecules in the MP-4K dataset. **a** Experimental melting point; **b** Melting point *vs* molecular weight. (colorbar: kernel-density estimate value)

For the design of liquid electrolytes, candidate solvents are expected to have low melting points to ensure that the system remains in the liquid state. Based on the MP-4K dataset, we also constructed a hard-voting ensemble model to classify whether the melting point of a molecule is less than or equal to 313.15 K. The model was built by using the same approach as for viscosity. The performance of the base classifiers and the ensemble model (denoted as XGB-MP-4K) is summarized in Table S7, and the importance of the top-10 most important features is shown in Fig. S11. As we can see, almost all evaluation metrics on the test sets exceed 0.9, indicating that the three classifiers achieve excellent performance. In addition, a new ensemble model (denoted as XGB-MP-592) was built based on 592 battery solvent-like molecules in MP-4K dataset. As shown in Table S8, the base classifiers exhibit remarkable

lower performance on the test sets than on the training sets, indicating poor generalization. Furthermore, their predictive performance on test positive samples is inferior to that of the XGB-MP-4K model. To evaluate the generalizability of models, melting points of 258 battery-like molecules were collected from the Bradley dataset,[11] which are not included in the MP-4K dataset and were used as an external dataset. It totally contains 126 negative samples and 132 positive samples. As can be seen from Table S7, the external test precision and recall of the XGB-MP-4K model for positive samples are 0.69 and 0.89, respectively, indicating that about 90% of true positive samples can be correctly identified. Moreover, these metrics are comparable to, or slightly outperform, those of the XGB-MP-592 model. (see Table S8). Therefore, the XGB-MP-4K model is selected as the final predictor for melting point.

Table S7: Performance of the base classifiers and the ensemble XGB-MP-4K model.

**model**	**dataset**	**class**	**precision**	**recall**	**F1-score**
base classifiers	3 training sets	0	0.99±0.01	0.99±0.01	0.99±0.01
		1	0.97±0.01	0.99±0.01	0.98±0.01
	3 test sets	0	0.94±0.01	0.95±0.01	0.95±0.01
		1	0.90±0.01	0.89±0.01	0.90±0.01
ensemble model	whole MP-4K dataset	0	0.99	0.98	0.99
		1	0.97	0.99	0.98
	592 battery	0	0.99	0.93	0.96
	solvent-like molecules	1	0.98	0.99	0.99
	258 battery	0	0.84	0.58	0.69
	solvent-like external molecules	1	0.69	0.89	0.78

Table S8: Performance of the base classifiers and the ensemble XGB-MP-592 model.

**model**	**dataset**	**class**	**precision**	**recall**	**F1-score**
base classifiers	3 training sets	0	0.95±0.06	0.99±0.01	0.97±0.03
		1	0.99±0.01	0.99±0.02	0.99±0.01
	3 test sets	0	0.61±0.13	0.70±0.12	0.65±0.13
		1	0.91±0.04	0.87±0.05	0.89±0.04
ensemble model	592 battery	0	0.97	0.93	0.98
	solvent-like molecules	1	0.98	0.99	0.99
	258 battery	0	0.78	0.66	0.71
	solvent-like external molecules	1	0.72	0.82	0.76

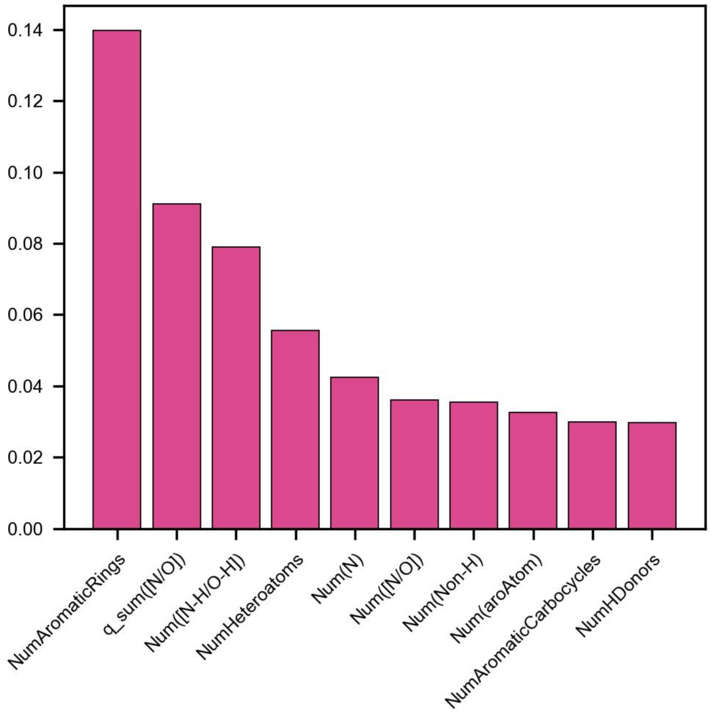

Figure S11: Importance of the top-10 features of the XGB-MP-4K ensemble model for melting point.

2.4 ML models for donor number

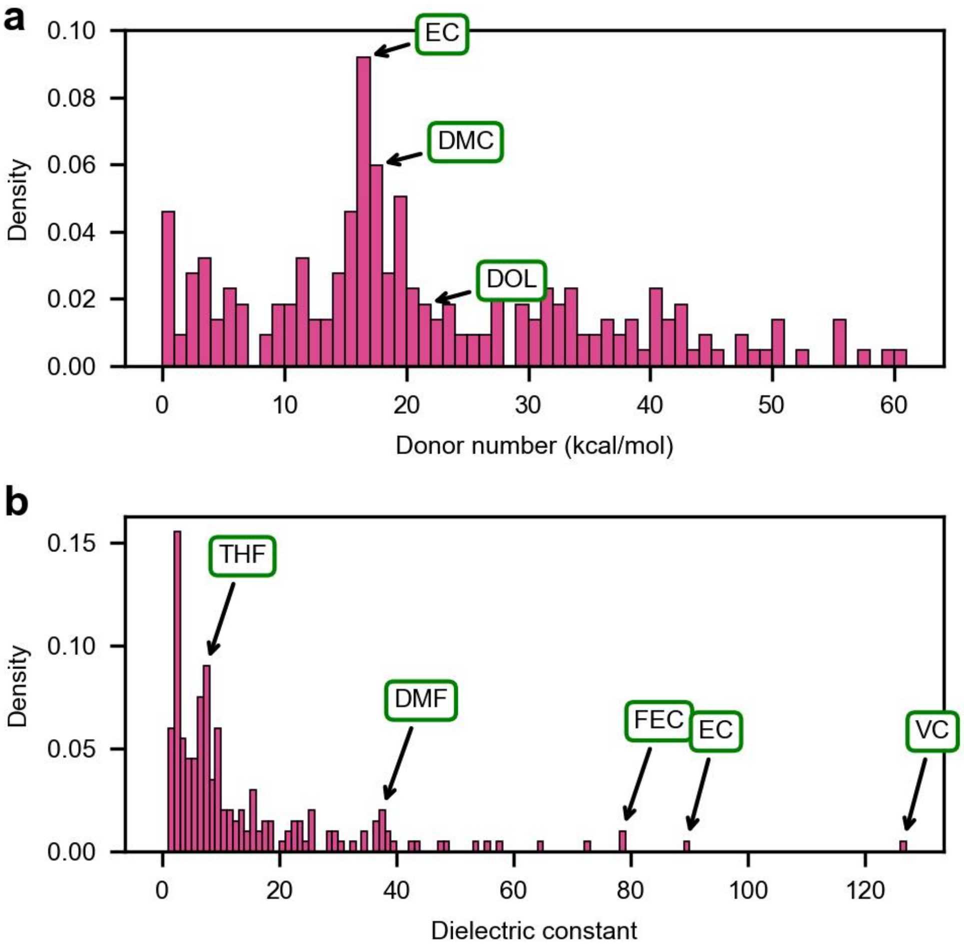

Figure S12: Distribution of experimental donor numbers in the DN-218 dataset and dielectric constants in the DC-200 dataset. **a** Experimental donor numbers; **b** Experimental dielectric constants.

Miranda-Quintana and Smiatek[12] derived the following expression for the reaction energy (e.g. the donor number) from the conceptual DFT.

$$\begin{aligned}\Delta E^{\gamma_{\mathrm{SbCl_5}},\gamma_{\mathrm{S}},\xi_{\mathrm{SbCl_5}},\xi_{\mathrm{S}}} =& -\frac{1}{8}\frac{\left(I_{\mathrm{SbCl_5}}+A_{\mathrm{SbCl_5}}-\left(I_{\mathrm{S}}+A_{\mathrm{S}}\right)\right)^2}{I_{\mathrm{SbCl_5}}-A_{\mathrm{SbCl_5}}+I_{\mathrm{S}}-A_{\mathrm{S}}} \\ &+\frac{1}{2\xi_{\mathrm{SbCl_5}}\left(1+\gamma_{\mathrm{SbCl_5}}\right)^2}\frac{\left(\gamma_{\mathrm{SbCl_5}}\left(I_{\mathrm{SbCl_5}}-A_{\mathrm{DCE}}\right)-I_{\mathrm{DCE}}+A_{\mathrm{SbCl}}\right)^2}{I_{\mathrm{SbCl_5}}-A_{\mathrm{SbCl_5}}+I_{\mathrm{DCE}}-A_{\mathrm{DCE}}} \\ &+\frac{1}{2\xi_{\mathrm{S}}\left(1+\gamma_{\mathrm{S}}\right)^2}\frac{\left(\gamma_{\mathrm{S}}\left(I_{\mathrm{S}}-A_{\mathrm{DCE}}\right)-I_{\mathrm{DCE}}+A_{\mathrm{S}}\right)^2}{I_{\mathrm{S}}-A_{\mathrm{S}}+I_{\mathrm{DCE}}-A_{\mathrm{DCE}}}\end{aligned} \quad \text{(S1)}$$

, where γ and ξ are empirical perturbation parameters which can be approximated as 1 to simplify the equation; I and A are the negative values of the HOMO and LUMO energies, respectively. After adding the ΔE into the molecular features in Table S4, we built an ensemble regressor model to predict the donor number of given molecules. The ensemble model includes three multi-linear regression (MLR)-based learners trained on different random splits of the dataset (training:test = 9:1). Before training the MLR-based regressor, a feature selection procedure was applied as follows: First, the normalized features with a lower variance than 0.01 were removed. Second, feature pairs with a Pearson correlation coefficient larger than 0.9 were filtered out, and the one in each pair has a relatively lower Pearson correlation coefficient with the training labels was eliminated. Third, the remaining features with a Pearson correlation coefficient with training labels smaller than 0.1 were removed. Forth, the final selected features were determined by a forward selection based on a default MLR model and the RMSE score. The ΔE feature was kept during the whole selection process. The performance of the ensemble model (denoted as MLR-DN-218) on the whole DN-218 dataset is shown in Fig. S13c, and the top-10 most important features were demonstrated in Fig. S13d.

To test whether the ensemble models trained on battery solvent-like molecules perform better than that on the entire DN-218, we applied the same criteria used to construct Bat-SLM and filtered out 128 battery solvent-like molecules from the DN-218 dataset. Then, a new ensemble model (denoted as MLR-DN-128) was built only on these 128 molecules. Its predictive performance and the corresponding permutation feature importance are illustrated in Fig. S14b and c. As one can see, its MAE (3.26 kcal/mol) on these 128 molecules is no significant improvement to that of the MLR-DN-218 model on the same molecules (3.85 kcal/mol in Fig. S14a). Considering that a larger training dataset typically results in a more generalized model, we decided to utilize the MLR-DN-218 ensemble model as the predictor of donor numbers.

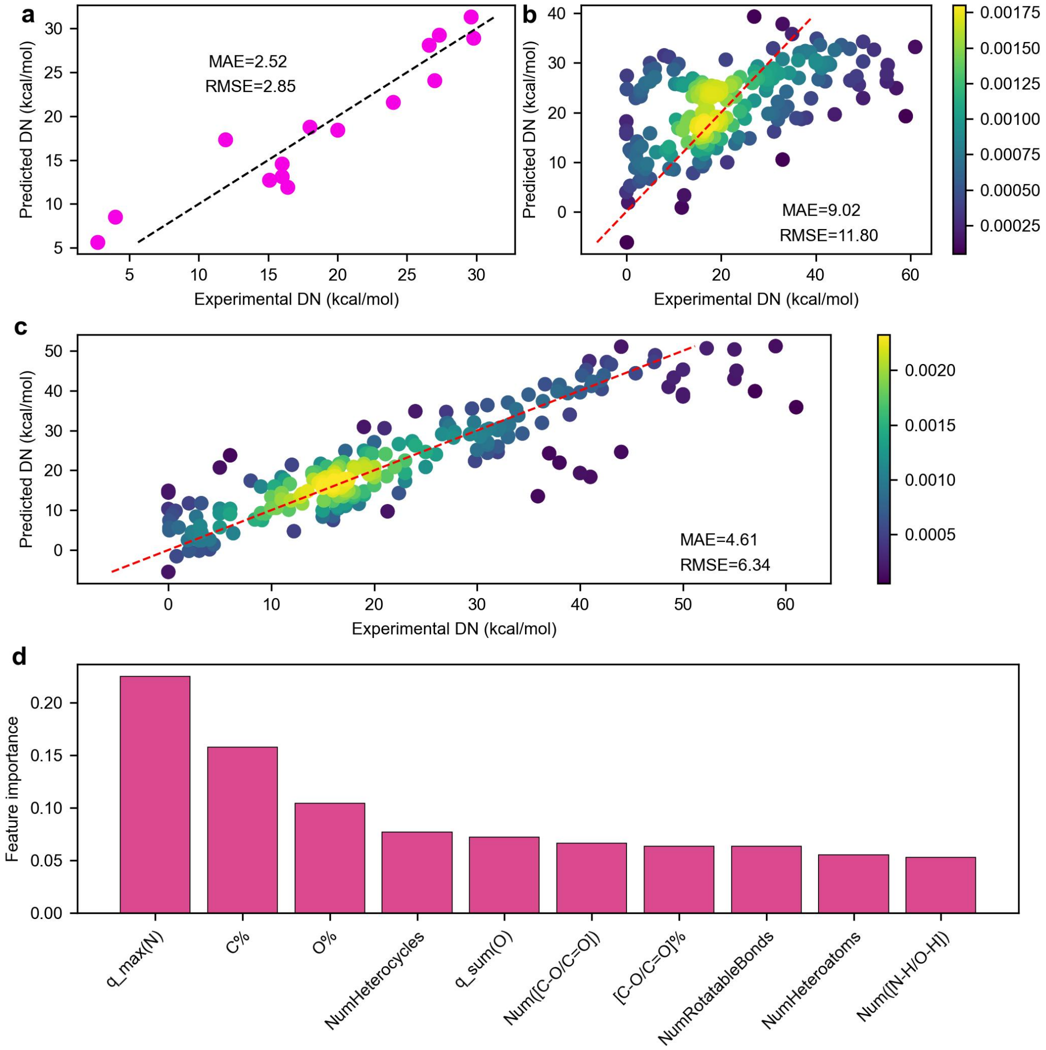

Figure S13: Performance of linear regression models for donor number (DN) and permutation feature importance. **a** The single linear regression model between donor number and ΔE computed at the ωB97X-V/def2-TZVPPD/SMD(ε=18.5) level for the 15 solvents demonstrated in ref. 10; **b** Performance of the single linear regression model on the whole DN-218 dataset; **c** Performance of the MLR-DN-218 ensemble model on the whole DN-218 dataset; **d** Permutation importance of the top-10 features of the MLR-DN-218 model. (color bar: kernel-density estimate value)

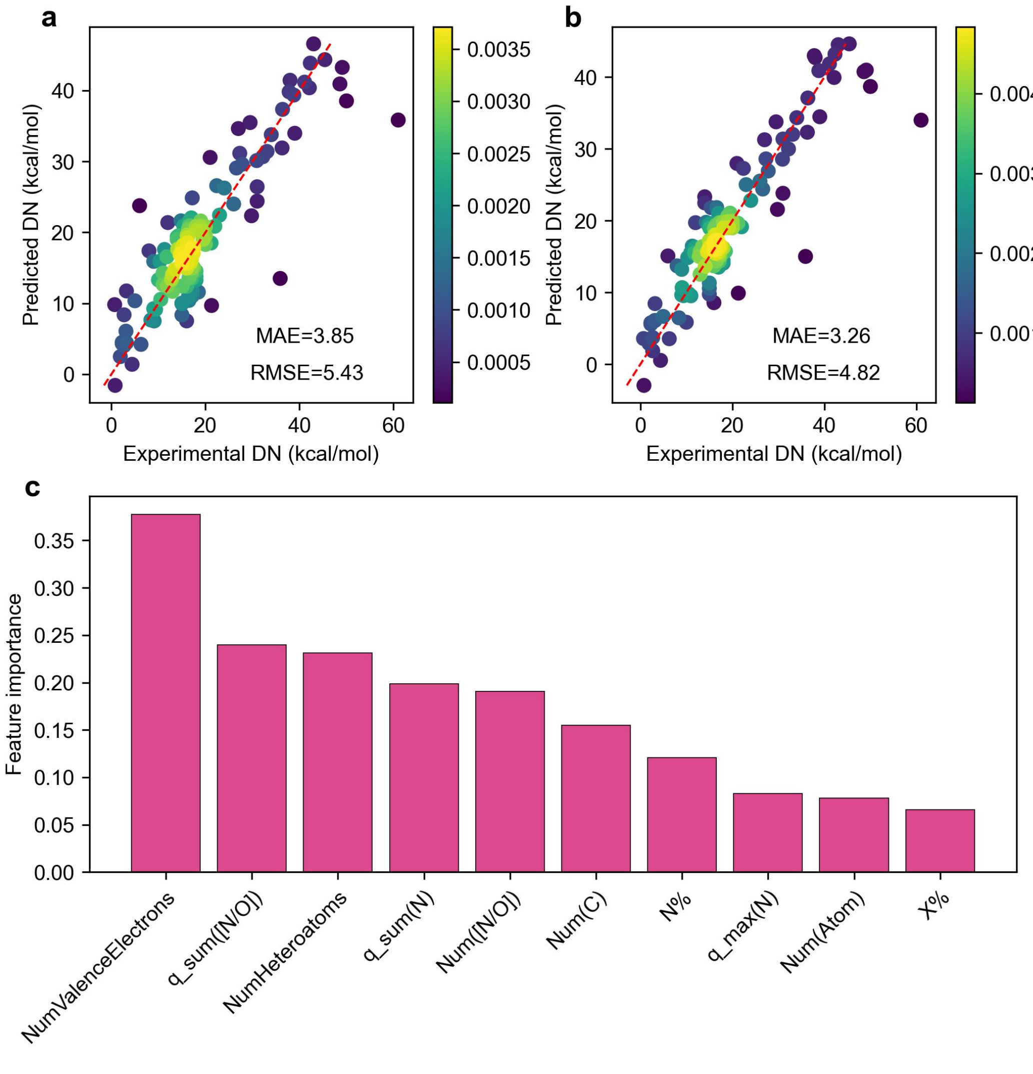

Figure S14: Performance of MLR-based ensemble models for the donor numbers of 128 battery solvent-like molecules and the permutation feature importance. **a** Performance of the MLR-DN-218 ensemble model; **b** Performance of the MLR-DN-128 ensemble model; **c** Permutation importance of the top-10 features of the MLR-DN-128 model.

2.5 ML models for dielectric constant

As described in the main text, the squared molecular dipole moments exhibit an approximately linear relationship with the corresponding dielectric constants. To achieve accurate dielectric constant prediction, two separate models were constructed based on different subsets of the DC-200 dataset. The first is a linear fitting model between the squared dipole moments and dielectric constants for molecules with dipole moments $\leq$ 4.0 Debye. The second model is an MLR-based ensemble model developed to predict the coefficient of this linear relationship for molecules with dipole moments $>$ 4.0 Debye. The ensemble model was constructed using the same strategy as that applied for donor number prediction. The combination of these two models is denoted as LF-MLR-DC-200. Considering that only 138 molecules in the DC-200 dataset satisfy the same selection criteria used for constructing Batt-SLM, two additional models (denoted as LF-MLR-DC-138) were further developed based on these 138 molecules. As shown in Fig. S15, the performance of LF-MLR-DC-138 on these 138 molecules yields an MAE of 4.16 and an RMSE of 6.94, showing no significant improvement over LF-MLR-DC-200 evaluated on the same subset. Therefore, LF-MLR-DC-200 was selected as the final model for dielectric constant prediction.

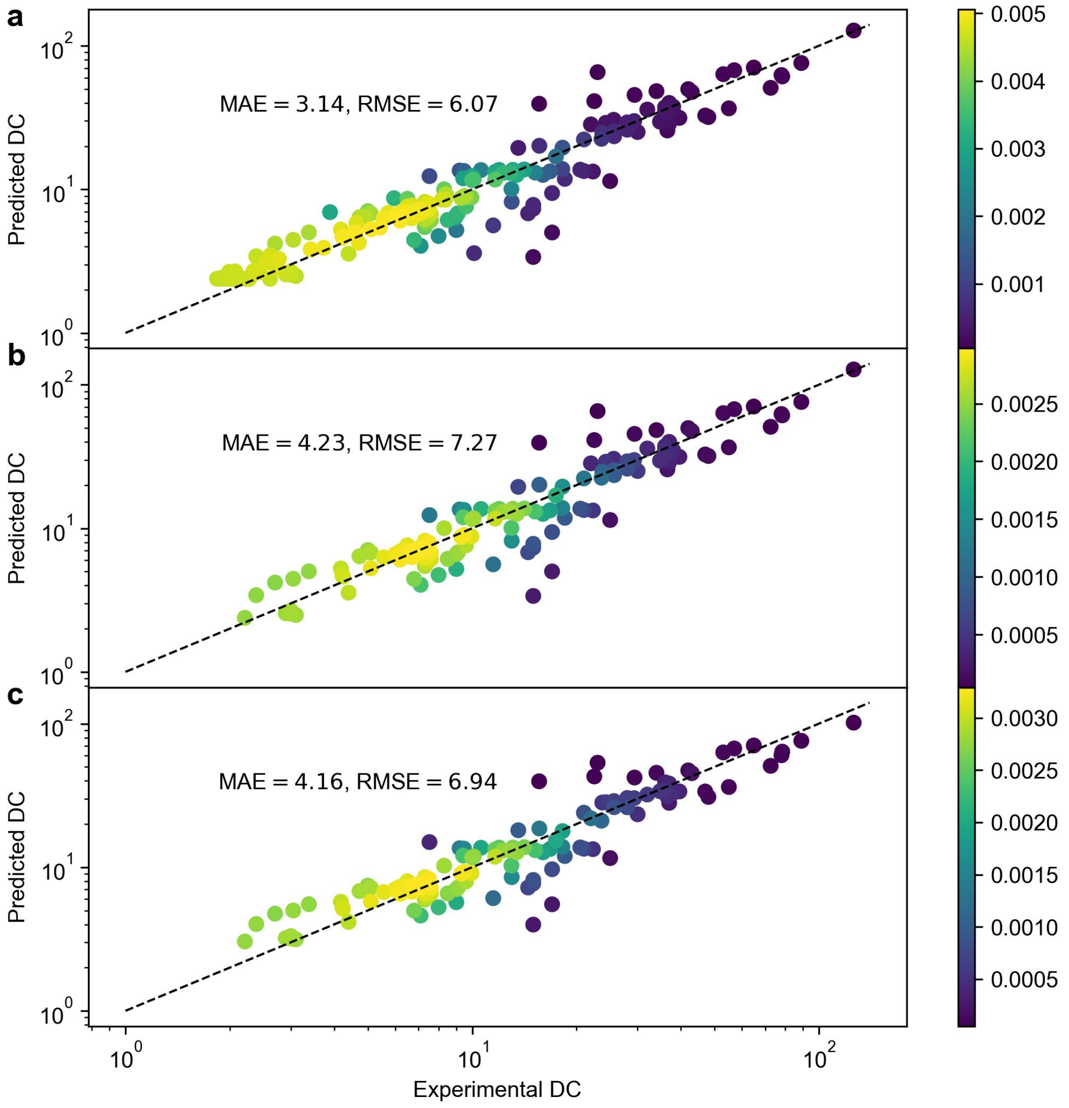

Figure S15: Performance of the fitted linear models on dielectric constants. **a** Performance of the LF-MLR-DC-200 on the whole DC-200 dataset; **b** Performance of the LF-MLR-DC-200 on the 138 battery solvent-like molecules; **c** Performance of the LF-MLR-DC-138 on the 138 battery solvent-like molecules. (colorbar: kernel-density estimate value)

3 Fine-tuning prior models in the rediscovery of the F/P-holdout

For the construction of a dataset for training the prior II models, the reference molecules for Tanimoto similarity in the elsewhere replaced by the known battery solvents without F and P atoms, and all molecules in the F/P-holdout set were excluded. Moreover, when resampling the PubChem molecules, the count of large molecules were reduced to 0.5 times of the count of small molecules, ensuring that the new dataset is comparable in size (117,372) to that of Batt-SLM (115,756).

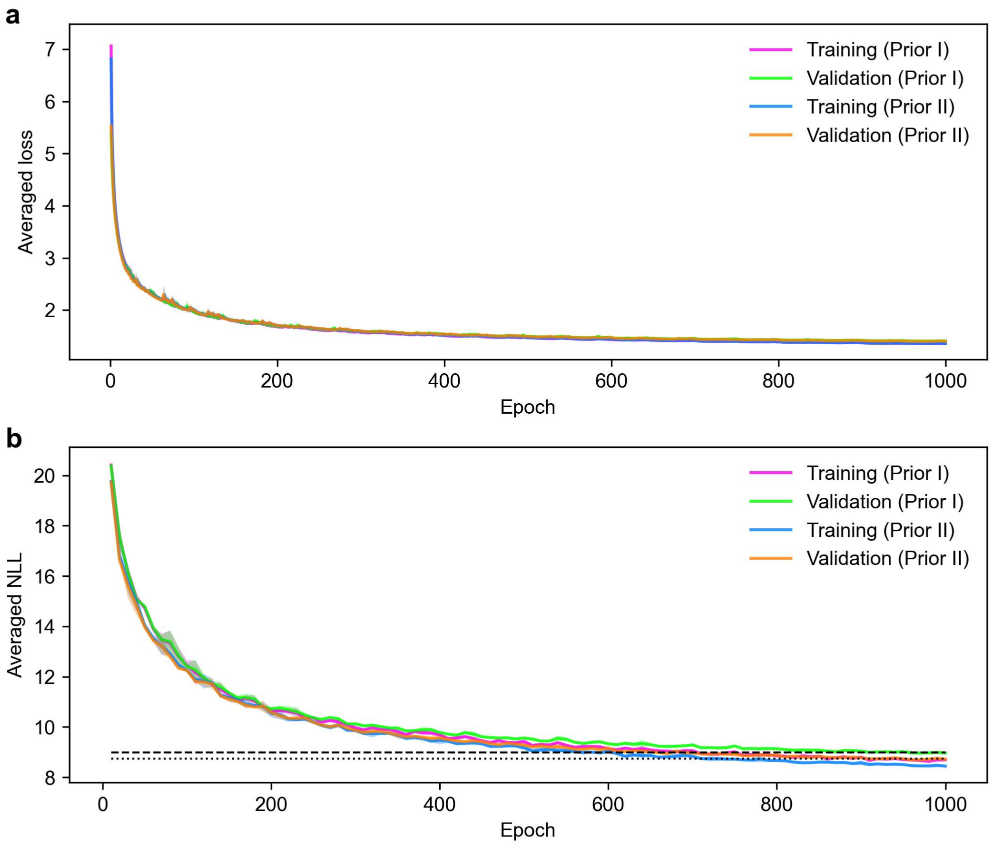

Figure S16: Training and validation losses, as well as averaged negative log-likelihood (NLL) taking the correct actions for per molecule of priors I and II. **a** Training and validation curves ; **b** Averaged NLL on the training and validation sets. The dashed and dotted lines represent the average test NLLs for prior I and prior II after 1000 epochs, respectively. The standard deviations obtained from 3 independent prior models are shown in transparent colors.

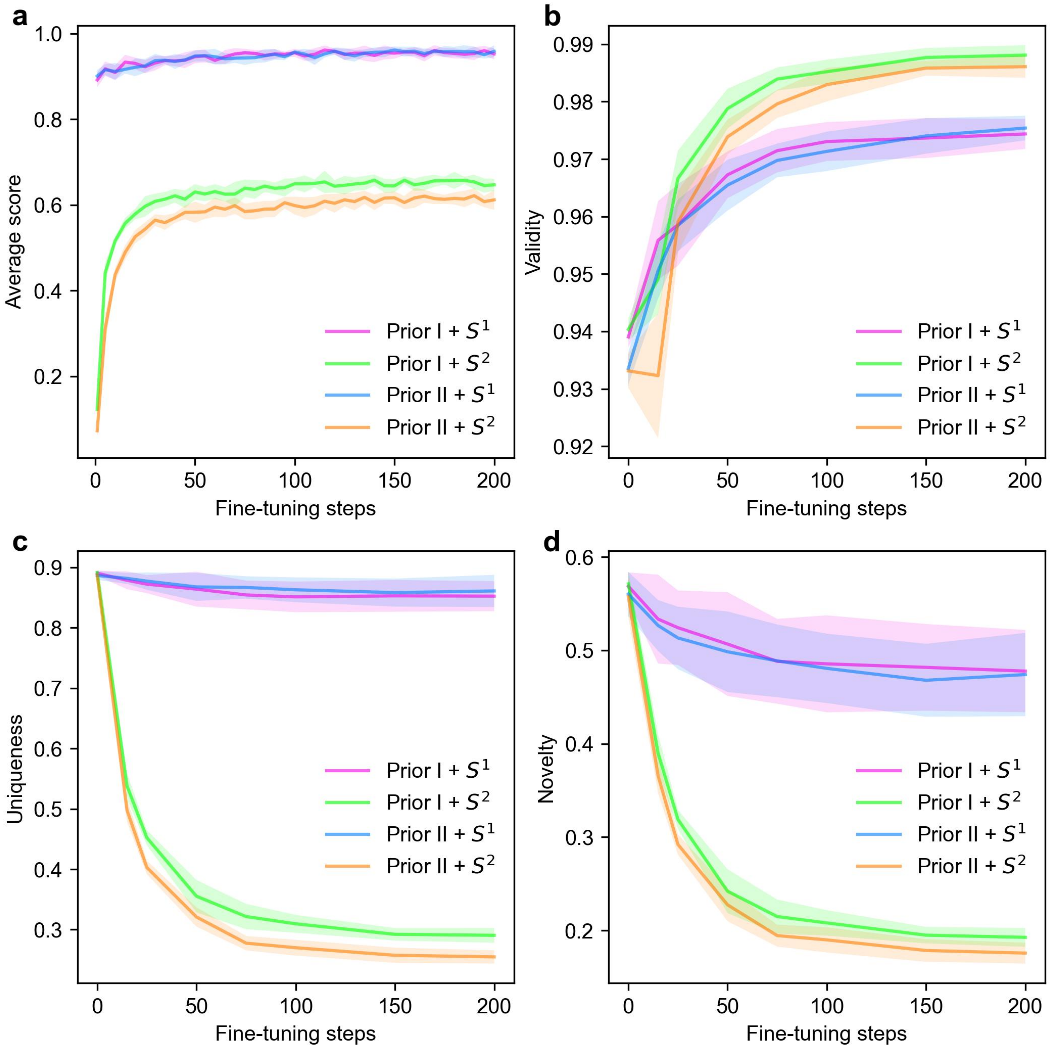

Figure S17: Fine-tuning validation average score and generative criteria estimated by 20,000 generated molecules of agent models from prior I and prior II combining with $S^1(G)$ and $S^2(G)$. **a** Fine-tuning validation average score; **b** Validity; **c** Uniqueness; **d** Novelty. The standard deviations obtained from 9 agent models are shown in transparent colors. Validity means the rate of valid molecules evaluated by RDKit among all generated molecules; Uniqueness means the rate of valid and unique molecules among all generated molecules; Novelty means the rate of valid, unique and new (with respect to corresponding Batt-SLM) molecules among all generated molecules.

4 Optimization of the scoring functions in reinforcement learning

Based on the rediscovery test results, the scoring function has a significant effect on the effectiveness of fine-tuning process. After given the concerned properties, it is also essential to explore how different mathematical types of scoring components affect the generative performance. Since the mathematical expressions of binary components are trivial, here we only focus on the continuous ones. In the case of discovering fluorinated solvents with low DN, the generated molecules are expected have a predicted $E_{\text{ox}}^{\text{Li}^+/\text{Li}} > 5.0$ V and a DN $<$ 10.0 kcal/mol. For the former, a straightforward normalized component $S_{\text{r},2} = E_{\text{ox}}^{\text{Li}^+/\text{Li}}/5.0$ if $E_{\text{red}}^{\text{Li}^+/\text{Li}} < 0$ is a reasonable option. However, the normalized format is unsuitable for the latter because of positive constraint of scores and the decreasing trend of scores as the DN increased. Instead, a simple exponential function-based component $S_{\text{dn},2} = \exp(1.0 - \text{DN}/10.0)$ is a possible choice. Combining above components with other properties, a suitable scoring function $S^3(G)$ was designed:

$$S^3(G) = \sqrt[6]{S_{\text{f},2}(G) \times S_{\text{s},2}(G) \times S_{\text{v}}(G) \times S_{\text{mp}}(G) \times S_{\text{dn},2}(G) \times S_{\text{r},2}(G)} \quad \text{(S2)}$$

Beyond the above simple formats of $E_{\text{ox}}^{\text{Li}^+/\text{Li}}$ and DN, another concern is their different weights within the scoring function. Mathematically speaking, the largest values of $S_{r,2}$ and $S_{\text{dn},2}$ is infinity and 2.72. It indicates that their contributions to the fine-tuning score are significantly different, which may lead to over-fine-tuning the agent to only one specific property. In this context, we anticipated that the all component values would fall within the range of 0 to 1. Since the binary components and the Tanimoto similarity-based $S_{\text{s},2}$ have already fulfilled, we only focus on the $E_{\text{ox}}^{\text{Li}^+/\text{Li}}$ and DN components. Considering the logistic function is a widely used format and meets the above requirements, the S_{dn} and S_{r} components in the main text were designed. Then, another suitable scoring function $S^4(G)$

was obtained:

$$S^4(G) = \sqrt[6]{S_{\mathrm{f},2}(G) \times S_{\mathrm{s},2}(G) \times S_{\mathrm{v}}(G) \times S_{\mathrm{mp}}(G) \times S_{\mathrm{dn}}(G) \times S_{\mathrm{r}}(G)} \quad \text{(S3)}$$

The $S^3(G)$ and $S^4(G)$ with different β combining with prior I were then utilized to conduct the hit rate tests of novel fluorinated solvents with low donor number. The hit rate means the ratio of novel F/P-containing molecules which passed through the filters on Batt-SLM, as well as have a predicted viscosity $\leq$ 2.0 cP, a melting point $\leq$ 313.15 K, a donor number $<$ 10 kcal/mol, a $E_{\mathrm{red}}^{\mathrm{Li^+/Li}} < 0.0$ V, and a $E_{\mathrm{ox}}^{\mathrm{Li^+/Li}} > 5.0$ V among all generated molecules. As shown in Fig. S18, the $S^4(G)$ possess a much lower average score because of its lower uniqueness and novelty. Whereas, around 5% of generated molecules by it meet all the structural and property requirements, which is almost double the value of $S^3(G)$. Therefore, for the results on in the Main Text, we have chosen to use the logistic scoring components for describing the continuous properties, which is a better option to fine-tune the prior models. What is more, a decrease in β for the donor number component led to a higher hit rate, whereas the influence of β on the hit ratio of $E_{\mathrm{ox}}^{\mathrm{Li^+/Li}}$ is ignorable. This phenomenon may be caused by the difference between the averaged properties of generated molecules by initial prior I and the related target thresholds. The property with a larger difference favors a smaller β, resulting in a more gradual increase or decrease in the logistic function. As we can see from Fig. S19, the averaged $E_{\mathrm{ox}}^{\mathrm{Li^+/Li}}$ among 1,959 generated molecules is 5.21 V which is very close to the target 5.0 V, while the averaged DN is 19.92 kcal/mol which is much larger than 10.0 kcal/mol. Based on the above results, the temperature parameter β for $S_{\mathrm{dn}}(G)$, $S_{\mathrm{r}}(G)$, and $S_{\mathrm{dc}}(G)$ was set as 1.0, 100.0, and 1.0, respectively. It is worth noting that, the hit rate increased significantly from approximately 0.002 (fine-tuning steps = 0) to 0.066 in the best fine-tuned model (Fig. S18f), suggesting that the reinforcement learning strategy improves the hit rate by a factor of approximately 30.

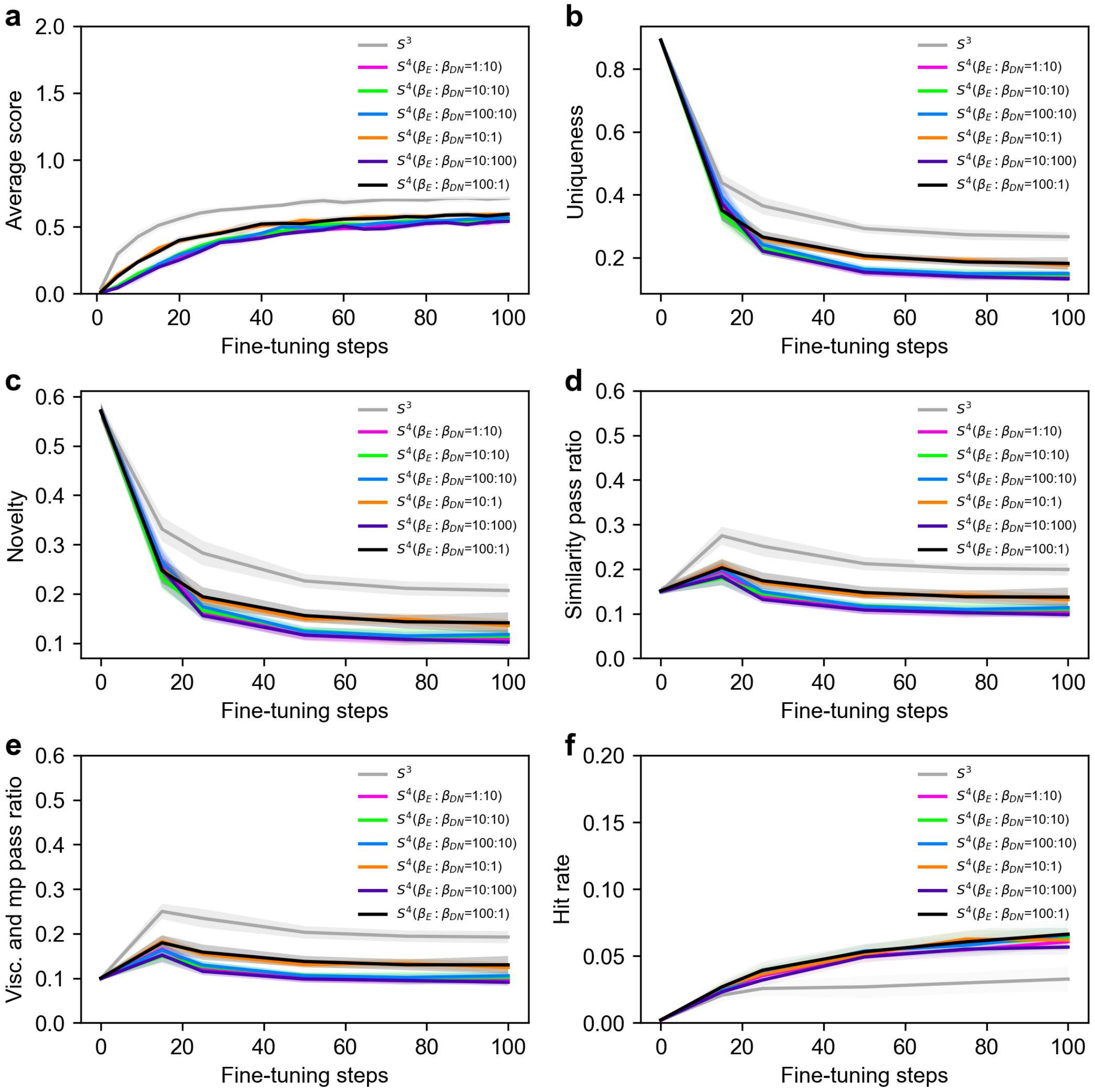

Figure S18: Fine-tuning validation average score and generative criteria estimated by 20,000 generated molecules of agent models from prior I combining with $S^3(G)$ and $S^4(G)$ with different temperature parameters in the logistic function. **a** Average score; **b** Uniqueness; **c** Novelty; **d** Ratio of novel molecules with a Tanimoto similarity > 0.2 to the F/P-holdout; **e** Ratio of molecules meet the above Tanimoto similarity requirement and with a predicted viscosity ≤ 2.0 cP as well as a melting point ≤ 313.15 K; **f** Ratio of molecules meet the above requirements and with a $E_{\mathrm{red}}^{\mathrm{Li^+/Li}} < 0.0\ V$, a $E_{\mathrm{ox}}^{\mathrm{Li^+/Li}} > 5.0\ V$, and a DN < 10 kcal/mol. The β_E and β_{DN} is the temperature parameter for $E_{\mathrm{ox}}^{\mathrm{Li^+/Li}}$ and donor number, respectively.

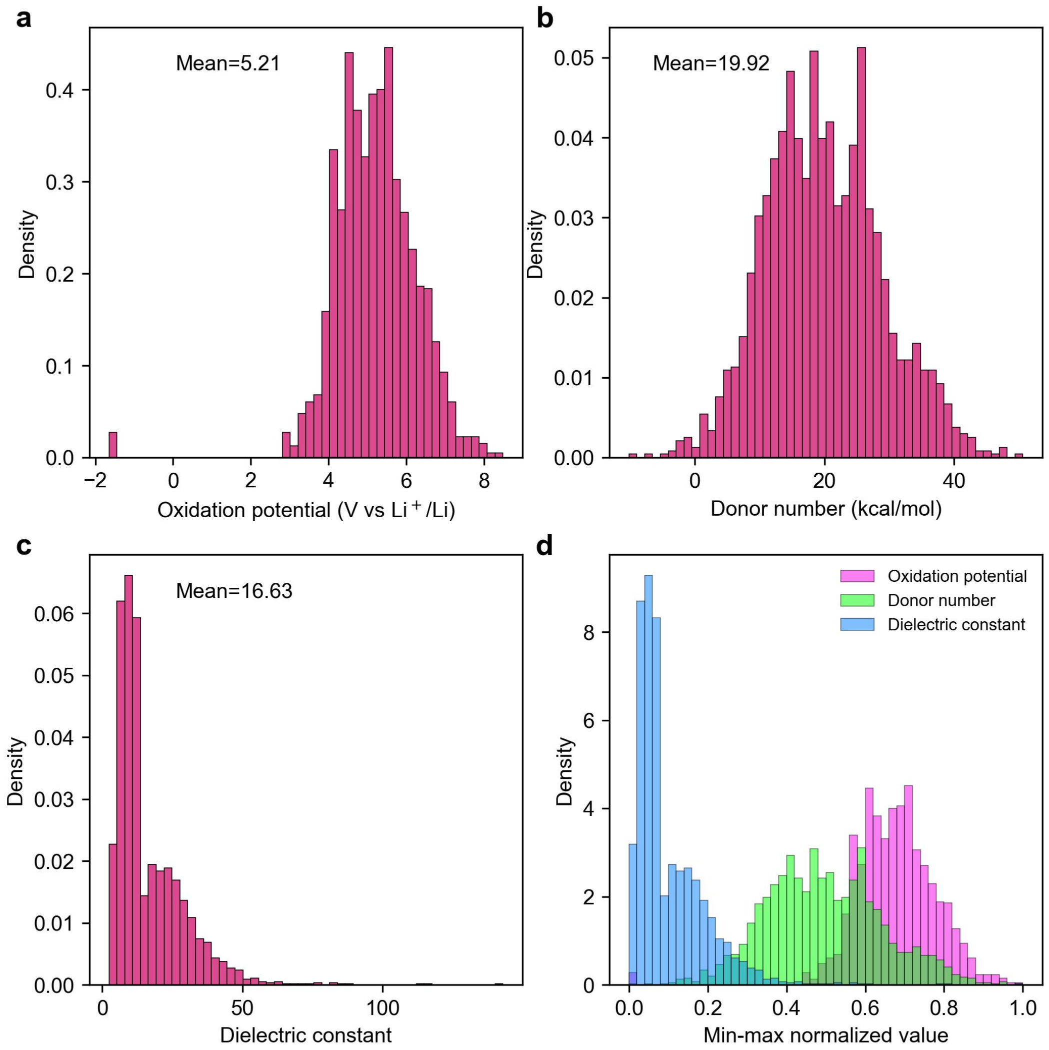

Figure S19: Distribution of predicted properties for 1,959 generated molecules by prior I before fine-tuning. **a** Oxidation potential; **b** Donor number; **c** Dielectric constant; **d** Min-max normalized properties.

The highest hit rate in Fig. S18 is merely about 6%, prompting further inquiry into the reasons behind this occurrence. Upon comparing the generative criteria of $S^2(G)$ (see Fig. S17) and $S^4(G)$, we observed a significant decrease in novelty (from 0.2 to 0.1). Considering this, the number of components in the scoring function is likely one reason for the decrease of hit rate. Thus, we designed two additional scoring functions by removing some components from $S^4(G)$:

$$S^5(G) = \sqrt[4]{S_{\mathrm{f},2}(G) \times S_{\mathrm{s},2}(G) \times S_{\mathrm{v}}(G) \times S_{\mathrm{mp}}(G)} \tag{S4}$$

$$S^6(G) = \sqrt[5]{S_{\mathrm{f},2}(G) \times S_{\mathrm{s},2}(G) \times S_{\mathrm{v}}(G) \times S_{\mathrm{mp}}(G) \times S_{\mathrm{r}}(G)} \tag{S5}$$

The comparison of generative criteria concerning $S^2(G)$, $S^5(G)$, $S^6(G)$, and $S^4(G)$ are shown in Fig. S20. It is evident that all criteria show a declining trend with the increase in the number of scoring components. For example, the hit rate of $S^5(G)$ are approximately 24%, while the value decreased to 18% for $S^6(G)$ and even plummeted to 6% for $S^4(G)$. Clearly, the $S_{\mathrm{dn}}(G)$ component in $S^4(G)$ contributed more significantly to the decrease of hit rate compared to $S_{\mathrm{r}}(G)$. Reflecting on the distribution of generated molecules by prior I before fine-tuning in Fig. S19, nearly half of molecules have a predicted oxidation potential $>$ 5.0, while only 12% molecules have a predicted donor number below 10 kcal/mol. The two findings mentioned above may indicate that the hit rate mainly relies on the component with a property requirement, which only a limited number of generated molecules can satisfy before fine-tuning. Despite the low hit rate of $S^4(G)$, it is still capable of generating sufficient novel molecules for the posterior verification.

Regarding to design non-fluorinated solvents, we also tested the hit rates of prior I and prior II combining with the following scoring function:

$$S^7(G) = \sqrt[6]{S_{\mathrm{f},3}(G) \times S_{\mathrm{s},1}(G) \times S_{\mathrm{v}}(G) \times S_{\mathrm{mp}}(G) \times S_{\mathrm{dc}}(G) \times S_{\mathrm{r}}(G)} \tag{S6}$$

As can be seen from Fig. S21, all of their generative criteria are comparable, which further

confirms that the scoring function has a more significant impact on the fine-tuning process compared to that of prior training sets. Therefore, the prior I was still used to generate the final candidates in the Main text.

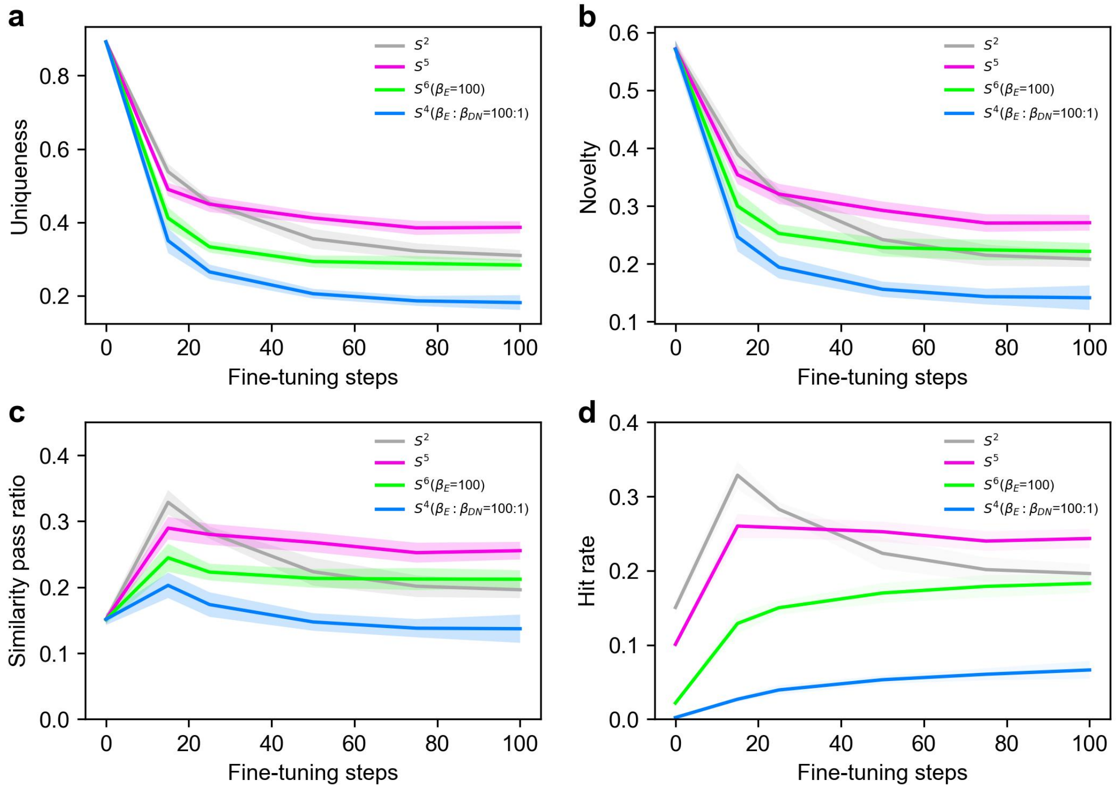

Figure S20: Generative criteria estimated by 20,000 generated molecules of agent models from prior I combining with $S^2(G)$, $S^5(G)$, $S^6(G)$, and $S^4(G)$, which containing 2,4,5,6 scoring components, respectively. **a** Uniqueness; **b** Novelty; **c** Ratio of novel molecules with a Tanimoto similarity > 0.2 to the F/P-holdout; **d** Ratio of molecules meet the above Tanimoto similarity and corresponding property requirements.

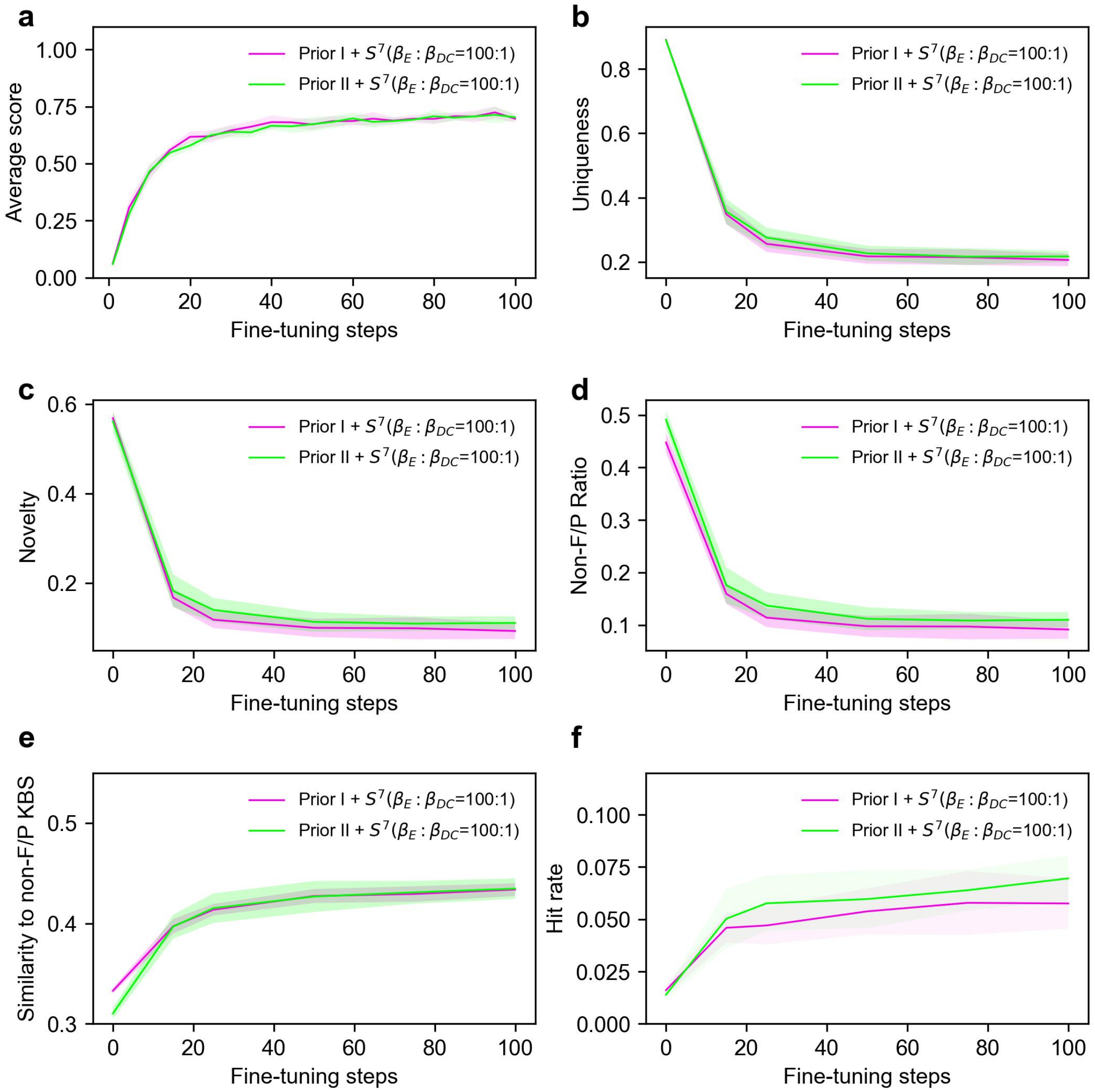

Figure S21: Fine-tuning validation average score and generative criteria estimated by 20,000 generated molecules of agent models from prior I and prior II combining with $S^7(G)$. **a** Average score; **b** Uniqueness; **c** Novelty; **d** Ratio of novel molecules without F/P atoms; **e** Ratio of novel molecules with a Tanimoto similarity $>$ 0.2 to the KBS-409 excluding the F/P-holdout; **f** Ratio of molecules meet the above Tanimoto similarity requirement and with a viscosity $\leq$ 2.0 cP as well as a melting point $\leq$ 313.15 K, a $E_{\text{red}}^{\text{Li}^+/\text{Li}} < 0.0\ V$, a $E_{\text{ox}}^{\text{Li}^+/\text{Li}} > 5.0\ V$, and a $\varepsilon < 10$. The β_E and β_{DC} is the temperature parameter for $E_{\text{ox}}^{\text{Li}^+/\text{Li}}$ and dielectric constant, respectively.

5 Construction of the posterior verification funnel

For the design of fluorine-containing solvents with low donor number, 180,000 SMILES were generated by the nine fine-tuned generators (after 100 fine-tuning steps with $S^4(G)$). In total, 7,188 unique molecules satisfy the DN requirement ($<$ 10.0 kcal/mol) but fail to meet the other property constraints. Then, 4,744 unique molecules satisfying all the structural and property requirements were identified. The relatively low hit rate (0.026), compared with that reported in Fig. S18, can be attributed to the presence of duplicate molecules from different generators. Among them, 2,691 have a SA score $\leq$ 4.0, a criterion chosen based on the KBS-409 (see Fig. S22), which can be considered as potentially synthesizable candidates.

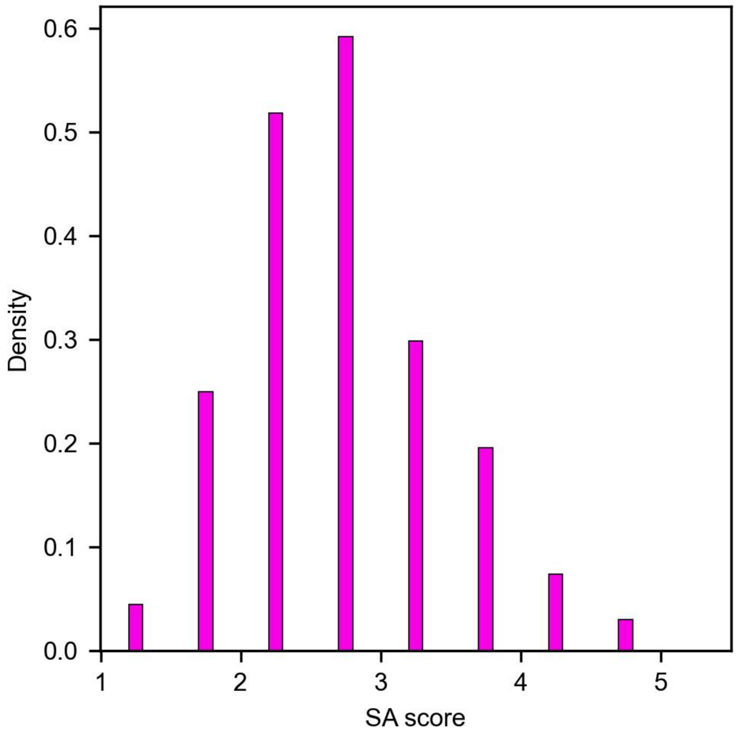

Figure S22: Distribution of SA scores for molecules in the KBS-409 dataset.

To ensure a high degree of novelty, we have included the following ingredients in the final step of the verification funnel. 50 candidates showing a maximum Tanimoto similarity greater than 0.7 to the F/P holdout set were excluded. Taking the potential per- and polyfluoroalkyl substances (PFAS) into consideration, 2,415 candidates containing fewer than seven fluorine

atoms were retained. Thereafter, these candidates were queried using the PubChem API[1] and annotated those found with their respective CIDs. Molecules with CIDs were further queried using the OpenAlex,[13] Semantic Scholar,[14] and PubMed[15] APIs, and those (N = 67) that had been used in battery systems were removed. High ring strain can hinder interactions with Li ions;[16] therefore, promising candidates are expected to have low ring strain energies (RSEs). To assess this, the recently developed ML-based workflow RSE_Atlas[17] was used to predict the RSEs of all candidate containing non-aromatic rings, including three-, four-, and five-membered structures. As shown in Fig. S23, the highest RSE of the solvents in KBS-409 is 20.24 kcal/mol. Therefore, candidates with predicted RSEs above 23 kcal/mol or those that failed in RSE prediction were further excluded.

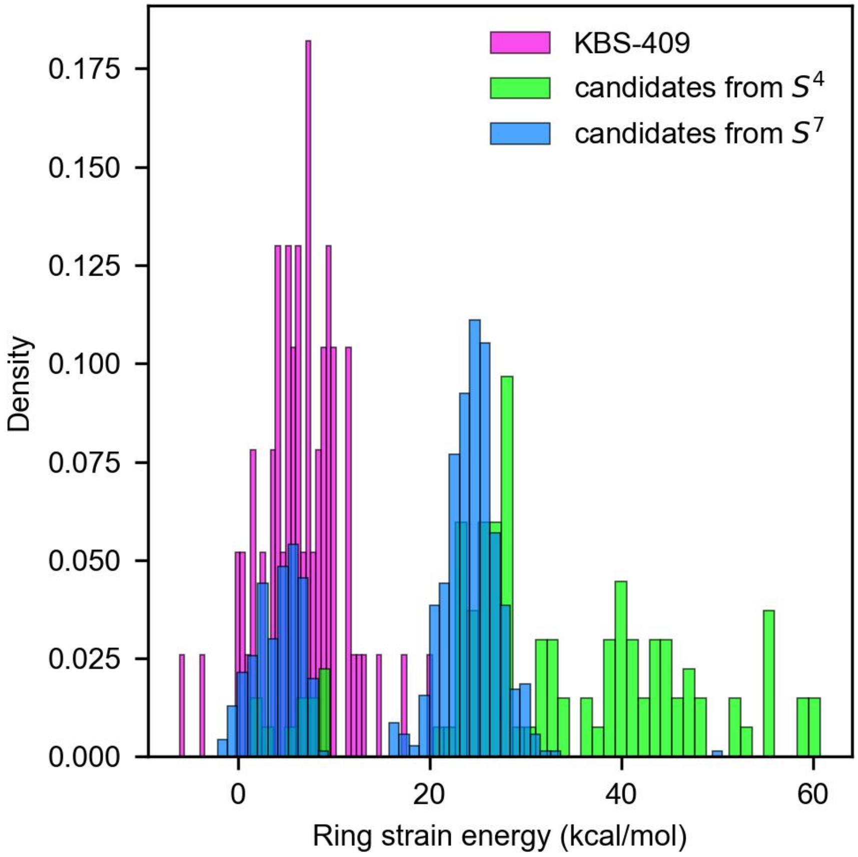

Figure S23: Distribution of predicted ring strain energies for 73 molecules with non-aromatic rings from the KBS-409 dataset, 113 candidates with non-aromatic rings of $S^4(G)$, and 667 candidates with non-aromatic rings of $S^7(G)$.

After the steps mentioned above, 2,174 candidates remained, which then underwent the following procedure to make a structurally diverse and novelty prioritized "promising set":

1. Grouping: Candidates were grouped according to their functional groups.

2. Initial selection: maximum Tanimoto similarities to the F/P holdout set were computed, and the molecule with a minimum Tanimoto similarity from each functional group was added to the "promising set".

3. Novelty prioritization: The maximum Tanimoto similarities of remaining candidates to those already in the promising set were calculated, and the molecule with the lowest Tanimoto similarity was added.

4. Iteration: Step 3 was repeated until the desired number of candidates was reached.

The above procedure were done for candidates molecules with and without CID separately to ensure an equal representativeness. In the end, this process yielded 64 candidates in the "promising set" , which were subsequently manually checked against the SciFinder database[18] to ensure that they are novel to battery applications. This leads to a final 30 candidates, which are reported in Figure 6 in the Main Tex and are also summarized in Table S10.

Before constructing the posterior verification funnel for non-fluorinated solvents, we evaluated the generalizability of CPI using both random splits and an external test set. For the random split evaluation, the CPI dataset was randomly divided into training and test sets (8:2) while preserving the distribution of functional groups. For the external test, an independent test set was constructed using 6 newly collected WSE solvents/additives from the literature (i.e., MEK,[19] 2Me-DOL,[20] DIGDBE,[21] HME,[22] MTBE,[23] and OTA[24]) and 9 non-fluorinated CDE solvents from KBS-406, none of which were included in the original CPI dataset. As shown in Table S9, in both the random split evaluations and external test, CPI achieved a recall of 1.0 for WSE solvents, while correctly identifying more than 88% CDE solvents. The lower precision for class 0 indicates that a small number of CDE solvents were misclassified as WSE.

Table S9: Performance of CPI on the 3 randomly split test sets and the external test set. (0: WSE; 1: CDE). The 9 CDE solvents in the external test set are dimethylacetamide (DMA), dimethylformamide (DMF), diethylformamide (DEF), methyl propyl carbonate (MPC), ethyl propyl carbonate (EPC), ethyl butyrate (EB), γ-butyrolactone (GBL), methyl formate (MF) and ethyl formate (EF).

**dataset**	**class**	**precision**	**recall**	**F1-score**
3 random splits	0	0.94 ± 0.08	1.00 ± 0.00	0.97 ± 0.04
	1	1.00 ± 0.00	0.92 ± 0.12	0.95 ± 0.07
15 external battery solvents/additives	0	0.86	1.00	0.92
	1	1.00	0.89	0.94

A similar verification funnel was applied to the discovery of non-fluorinated solvents with low dielectric constant, where a total of 180,000 SMILES were generated by the nine fine-tuned generators (after 100 RL steps with $S^7(G)$). In total, 7,832 unique molecules satisfy the DC requirement ($<$ 10.0) but fail to meet the other property constraints. Among them, 4,647 distinct SMILES met all the structural and property requirements (including CPIs $<$0.5), and 4,471 were identified as potentially synthesizable samples (SA score $\leq$ 4.0). Then, 75 candidates with a maximum Tanimoto similarity greater than 0.7 to solvents without fluorine in KBS-409 were excluded, and those with CPI values exceeding 0.5 (N=548) were further removed. The remaining molecules were queried, filtered based on their RSEs ($<$ 23 kcal/mol), and used to get the structurally diverse and novelty prioritized "promising set", following the similar procedure described above. This leads to a final 30 candidates which are reported in Figure 8 in the Main Text and also listed in Table S11.

Table S10: Synthesizable F-containing candidates with a predicted donor number $<$ 10.0 kcal/mol that have not been used in battery systems, and their predicted properties.

Category	CID	structure	$E_{red}^{Li^+/Li}$	$E_{ox}^{Li^+/Li}$	DN	ε	SA Score
Amides	–	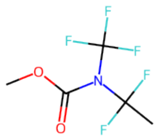	-0.13	6.89	7.79	8.52	3.27

Table S10: Continue.

Category	CID	structure	$E_{\text{red}}^{\text{Li}^+/\text{Li}}$	$E_{\text{ox}}^{\text{Li}^+/\text{Li}}$	DN	ε	SA Score
Ethers	–	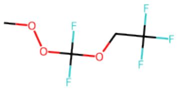	-0.14	6.54	4.02	21.58	3.52
Ethers	–	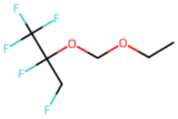	-0.22	6.17	8.89	10.30	3.84
Ethers	145814550	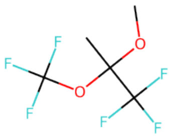	-0.14	6.51	3.97	11.14	3.65
Ethers	–	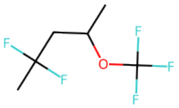	-0.28	7.06	8.50	9.32	3.63
Ethers	–	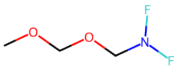	-0.20	5.98	7.34	12.23	3.89
Ethers	173255145	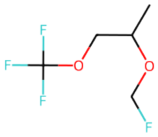	-0.23	6.08	9.23	8.49	3.78
Ethers	–	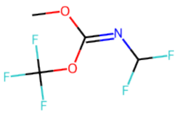	-0.06	6.71	8.44	29.38	3.78
Ethers	135383201	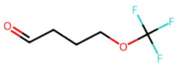	-0.01	5.42	9.98	9.81	2.76
Formates	–	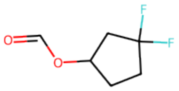	-0.20	6.42	7.50	13.33	3.81

Table S10: Continue.

Category	CID	structure	$E_{\mathrm{red}}^{\mathrm{Li}^+/\mathrm{Li}}$	$E_{\mathrm{ox}}^{\mathrm{Li}^+/\mathrm{Li}}$	DN	ε	SA Score
Formates	57445145	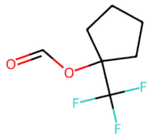	-0.15	6.54	6.37	3.08	3.34
Formates	13500160	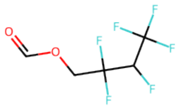	-0.06	6.78	2.29	4.27	3.85
Formates	–	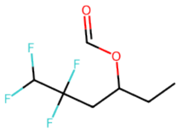	-0.18	6.42	9.65	13.51	3.89
Ketones	–	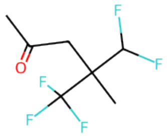	-0.04	5.47	9.97	21.28	3.61
Linear Carbonates	–	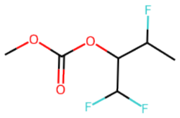	-0.14	6.93	7.70	7.23	3.92
Linear Carbonates	–	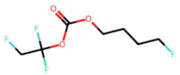	-0.15	7.03	6.94	18.39	3.12
Linear Carbonates	–	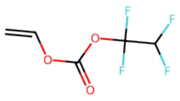	-0.09	5.55	2.06	8.01	3.50
Linear Carbonates	–	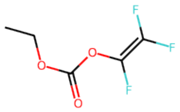	-0.01	5.43	4.47	11.81	3.22
Other Esters	175464861	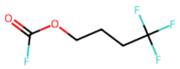	-0.03	7.15	4.94	24.74	2.81

Table S10: Continue.

Category	CID	structure	$E_{\mathrm{red}}^{\mathrm{Li^+/Li}}$	$E_{\mathrm{ox}}^{\mathrm{Li^+/Li}}$	DN	ε	SA Score
Other Esters	172738404	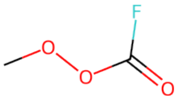	-0.13	6.84	7.22	10.85	3.68
Other Esters	69626812	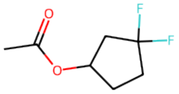	-0.22	6.14	8.86	17.40	3.18
Other Esters	–	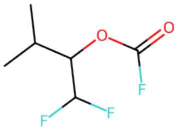	-0.07	7.22	8.63	17.96	3.83
Other Esters	168185092	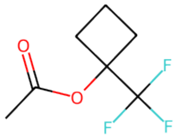	-0.25	6.25	7.15	5.79	2.86
Other Esters	–	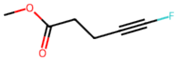	-0.11	5.60	8.65	11.31	3.28
Other Esters	–	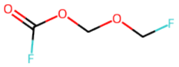	-0.12	6.97	6.13	12.48	3.76
Other Esters	–	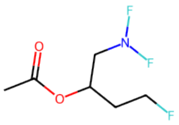	-0.16	6.30	7.21	27.07	4.00
Other Esters	24887638	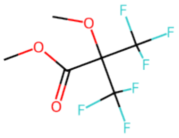	-0.11	6.60	3.07	13.45	3.06
Other Esters	–	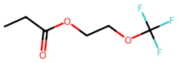	-0.19	6.20	8.84	7.66	2.41

Table S10: Continue.

Category	CID	structure	$E_{\mathrm{red}}^{\mathrm{Li^+/Li}}$	$E_{\mathrm{ox}}^{\mathrm{Li^+/Li}}$	DN	ε	SA Score
Other Esters	–	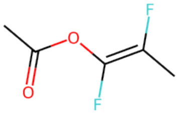	-0.12	5.04	8.65	33.25	3.55
Other Esters	–	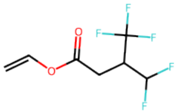	-0.02	5.31	5.50	13.71	3.94

Table S11: Synthesizable candidates without F atoms with a predicted dielectric constant < 10.0 and a chelation propensity index < 0.5 that have not been used in battery systems, and their predicted properties.

Category	CID	structure	$E_{\mathrm{red}}^{\mathrm{Li^+/Li}}$	$E_{\mathrm{ox}}^{\mathrm{Li^+/Li}}$	DN	ε	SA Score
Ethers	166973575	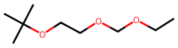	-0.12	5.23	17.45	6.81	2.49
Ethers	–	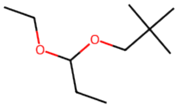	-0.40	5.27	18.66	7.49	3.20
Ethers	–	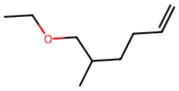	-0.33	5.10	20.54	4.41	2.96
Ethers	87508938	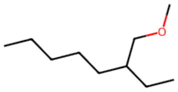	-0.35	5.15	22.63	4.34	2.47
Ethers	–	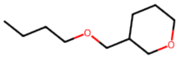	-0.28	5.07	22.80	5.19	2.61
Ethers	–	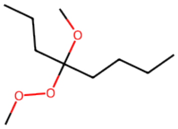	-0.28	5.30	19.90	4.71	3.69
Ethers	59190025	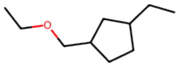	-0.39	5.14	19.28	4.19	2.96
Ethers	–	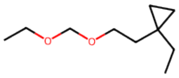	-0.26	5.33	18.99	6.24	2.82
Ethers	–	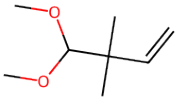	-0.33	5.02	18.44	9.47	3.39

Table S11: Continue.

Category	CID	structure	$E_{\text{red}}^{\text{Li}^+/\text{Li}}$	$E_{\text{ox}}^{\text{Li}^+/\text{Li}}$	DN	ε	SA Score
Ethers	–	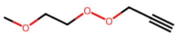	-0.22	5.43	13.84	5.33	3.32
Ethers	88975208	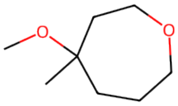	-0.34	5.00	21.08	5.46	3.19
Ethers	132210531	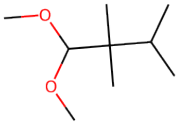	-0.32	5.34	20.51	8.16	3.08
Ethers	5314306	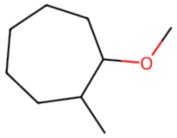	-0.31	5.03	17.71	4.41	2.76
Ethers	76713839	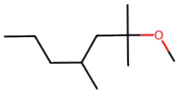	-0.44	5.01	22.58	4.56	2.98
Ethers	–	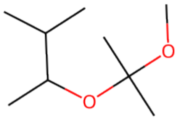	-0.37	5.19	20.48	5.00	3.42
Ethers	–	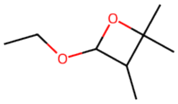	-0.31	5.40	21.72	4.03	3.71
Ethers	164846249	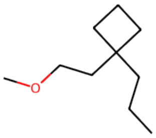	-0.30	5.11	20.01	4.42	2.48
Ethers	–	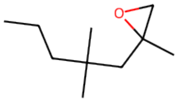	-0.29	5.44	24.71	6.85	3.61

Table S11: Continue.

Category	CID	structure	$E_{\text{red}}^{\text{Li}^+/\text{Li}}$	$E_{\text{ox}}^{\text{Li}^+/\text{Li}}$	DN	ε	SA Score
Ethers	–	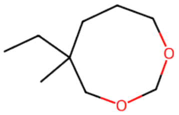	-0.29	5.36	20.32	2.90	3.45
Ethers	–	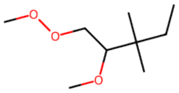	-0.26	5.19	20.08	6.69	3.71
Ethers	–	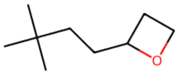	-0.30	5.01	21.82	6.60	2.83
Ethers	–	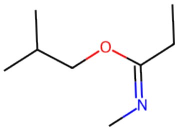	-0.27	5.06	30.12	8.01	3.16
Ethers	–	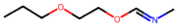	-0.25	5.20	28.93	4.20	3.16
Ethers	–	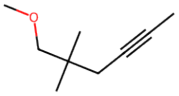	-0.43	5.15	18.08	4.27	3.31
Ethers	–	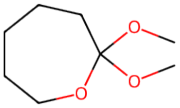	-0.29	5.56	18.60	7.61	3.10
Ethers	141852272	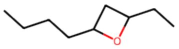	-0.27	5.02	24.17	6.53	3.01
Ethers	91749787	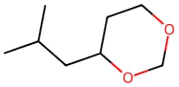	-0.25	5.25	20.84	5.22	3.18

Table S11: Continue.

Category	CID	structure	$E_{\mathrm{red}}^{\mathrm{Li^+/Li}}$	$E_{\mathrm{ox}}^{\mathrm{Li^+/Li}}$	DN	ε	SA Score
Other Esters	–	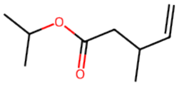	-0.01	5.15	16.23	7.58	3.08
Others	19381993	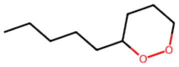	-0.29	5.18	21.85	9.74	3.25
Others	152051978	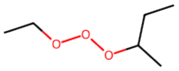	-0.27	5.82	16.95	8.19	3.74

6 Feasible candidates for experimental validation

The candidates listed in Figs. 6 and 8 in the Main Text were prioritized in terms of novelty. A different eye to look into the problem is to see what are commercially available and feasible for experimental validation.

To this end, we queried all new candidates that satisfy all the property and synthesizability constraints (2,691 for fluorine-containing molecules and 4,471 for non-fluorinated solvents, see the previous section for details) using the APIs of ChemSpace and MolPort websites.[25,26] Among them, 9 candidates in Figs. 6 and 8 in the Main Text are commercially available. They are summarized in Table S12. In addition, Table S12 includes 5 other candidates that are also purchasable with a price lower than 100 EUR/g while have been already reported in the battery literature.

Table S12: Commercially available candidates found on ChemSpace or MolPort websites. Gray rows represent the candidates that were discovered by GSDS but have been already reported in the battery literature.

Category	CID	structure	$E_{\mathrm{Red}}^{\mathrm{Li^+/Li}}$	$E_{\mathrm{Ox}}^{\mathrm{Li^+/Li}}$	DN	ε	Price (EUR/g)
Ethers	135383201	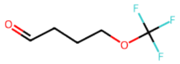	-0.01	5.42	9.98	9.81	1150
Formates	13500160	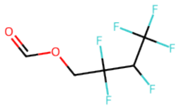	-0.06	6.78	2.29	4.27	–
Other Esters	69626812	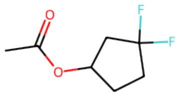	-0.22	6.14	8.86	17.40	–
Other Esters	24887638	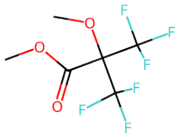	-0.11	6.60	3.07	13.45	908

Table S12: Continue.

Category	CID	structure	$E_{\mathrm{Red}}^{\mathrm{Li^+/Li}}$	$E_{\mathrm{Ox}}^{\mathrm{Li^+/Li}}$	DN	ε	Price (EUR/g)
Other Esters	–	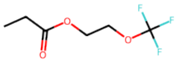	-0.19	6.20	8.84	7.66	1095
Ethers	–	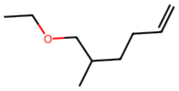	-0.33	5.10	20.54	4.41	1095
Ethers	–	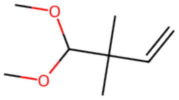	-0.33	5.02	18.44	9.47	1455
Ethers	132210531	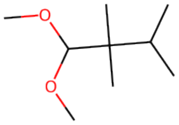	-0.32	5.34	20.51	8.16	1362
Other Esters	–	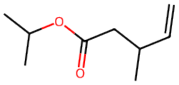	-0.01	5.15	16.23	7.58	704
Ethers	5206[27]	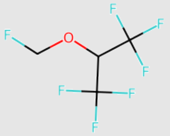	-0.19	7.32	1.70	7.62	21.44
Ethers	3733234[28]	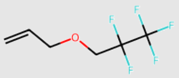	-0.21	5.45	8.58	10.14	90.41
Ethers	205999[29]	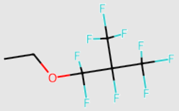	-0.14	7.53	1.56	12.28	0.36
Other Esters	7775[30]	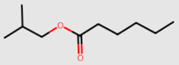	-0.14	5.94	18.20	6.07	0.41

Table S12: Continue.

Category	CID	structure	$E_{\text{Red}}^{\text{Li}^+/\text{Li}}$	$E_{\text{Ox}}^{\text{Li}^+/\text{Li}}$	DN	ε	Price (EUR/g)
Ethers	83755[31]	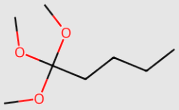	-0.32	5.68	18.30	4.65	15.84

References

1. Kim, S.; Chen, J.; Cheng, T.; Gindulyte, A.; He, J.; He, S.; Li, Q.; Shoemaker, B. A.; Thiessen, P. A.; Yu, B.; Zaslavsky, L.; Zhang, J.; Bolton, E. E. PubChem 2025 Update. *Nucleic Acids Res.* **2025**, *53*, D1516–D1525.

2. Rogers, D. J.; Tanimoto, T. T. A Computer Program for Classifying Plants: The Computer is Programmed to Simulate the Taxonomic Process of Comparing Each Case with Every Other Case. *Science* **1960**, *132*, 1115–1118.

3. Morgan, H. L. The Generation of A Unique Machine Description for Chemical Structures-A Technique Developed at Chemical Abstracts Service. *J. Chem. Doc.* **1965**, *5*, 107–113.

4. Halgren, T. A. Merck Molecular Force Field. I. Basis, Form, Scope, Parameterization, and Performance of MMFF94. *J. Comput. Chem.* **1996**, *17*, 490–519.

5. Halgren, T. A. Merck Molecular Force Field. II. MMFF94 Van Der Waals and Electrostatic Parameters for Intermolecular Interactions. *J. Comput. Chem.* **1996**, *17*, 520–552.

6. Halgren, T. A. Merck Molecular Force Field. III. Molecular Geometries and Vibrational Frequencies for MMFF94. *J. Comput. Chem.* **1996**, *17*, 553–586.

7. Kovács, D. P.; Moore, J. H.; Browning, N. J.; Batatia, I.; Horton, J. T.; Pu, Y.; Kapil, V.;

Witt, W. C.; Magdau, I.-B.; Cole, D. J.; Csányi, G. MACE-OFF: Short-Range Transferable Machine Learning Force Fields for Organic Molecules. *J. Am. Chem. Soc.* **2025**, *147*, 17598–17611.

8. Li, J.; Knijff, L.; Zhang, Z.-Y.; Andersson, L.; Zhang, C. PiNN: Equivariant Neural Network Suite for Modeling Electrochemical Systems. *J. Chem. Theory Comput.* **2025**, *21*, 1382–1395.

9. Wang, D.; He, T.; Wang, A.; Guo, K.; Avdeev, M.; Ouyang, C.; Chen, L.; Shi, S. A Thermodynamic Cycle-based Electrochemical Windows Database of 308 Electrolyte Solvents for Rechargeable Batteries. *Adv. Funct. Mater.* **2023**, *33*, 2212342.

10. Chew, A. K.; Sender, M.; Kaplan, Z.; Chandrasekaran, A.; Chief Elk, J.; Browning, A. R.; Kwak, H. S.; Halls, M. D.; Afzal, M. A. F. Advancing Material Property Prediction: Using Physics-Informed Machine Learning Models for Viscosity. *J. Cheminform.* **2024**, *16*, 31.

11. Bradley, J.-C.; Lang, A.; Williams, A. Jean-Claude Bradley Double Plus Good (highly curated and validated) Melting Point Dataset. **2014**, http://dx.doi.org/10.6084/m9.figshare.1031638, Accessed: 2026-05-27.

12. Miranda-Quintana, R. A.; Smiatek, J. Calculation of Donor Numbers: Computational Estimates for the Lewis Basicity of Solvents. *J. Mol. Liq.* **2021**, *322*, 114506.

13. Priem, J.; Piwowar, H.; Orr, R. OpenAlex: A Fully-Open Index of Scholarly Works, Authors, Venues, Institutions, and Concepts. *arXiv preprint arXiv:2205.01833* **2022**,

14. Kinney, R.; Anastasiades, C.; Authur, R.; Beltagy, I.; Bragg, J.; Buraczynski, A.; Cachola, I.; Candra, S.; Chandrasekhar, Y.; Cohan, A.; others The Semantic Scholar Open Data Platform. *arXiv preprint arXiv:2301.10140* **2023**,

15. National Center for Biotechnology Information (NCBI), USA PubMed / Entrez Programming Utilities (E-utilities) API. https://www.ncbi.nlm.nih.gov/home/develop/api/.

16. Choi, J.; Shin, K.-H.; Han, Y.-K. Origin of Li^+ Solvation Ability of Electrolyte Solvent: Ring Strain. *Materials* **2023**, *16*, 6995.

17. Liu, Z.; Vinskus, J.; Fu, Y.; Liu, P.; Noonan, K. J.; Isayev, O. Fast and Accurate Ring Strain Energy Predictions with Machine Learning and Application in Strain-Promoted Reactions. *JACS Au* **2025**, *5*, 4750–4761.

18. Gabrielson, S. W. SciFinder. *J. Med. Libr. Assoc.* **2018**, *106*, 588.

19. Shi, X.; Xie, J.; Wang, J.; Xie, S.; Yang, Z.; Lu, X. A Weakly Solvating Electrolyte towards Practical Rechargeable Aqueous Zinc-Ion Batteries. *Nat. Commun.* **2024**, *15*, 302.

20. Yang, Y.; Song, H.; Tu, H.; Ding, P.; Wu, G.; Liu, Z.; Gao, Y.; Wang, Z.; Xue, J.; Lu, S.; others Suppressing Solvent Co-Intercalation through Weakly Solvating Structure Regulation for Practical Li-Ion Sulfur Batteries. *Electrochim. Acta* **2025**, 147629.

21. Li, Z.; Chen, X.; Li, W.; Li, J.; Zhang, Y.; Lu, L.; Zhan, C.; Qiu, X. Tuning the End Alkyl Chain of the Ether Solvent to Stabilize the Electrode/Electrolyte Interfaces in the NCM-Li Battery. *ACS Appl. Mater. Interfaces* **2024**, *16*, 27429–27438.

22. Jin, T.; Li, X.-Y.; Zhao, M.; Feng, S.; Li, Z.; Chen, Z.-X.; Peng, H.-J.; Li, B.-Q.; Huang, J.-Q. Promoting the Rate Performances of Weakly Solvating Electrolyte-Based Lithium–Sulfur Batteries. *Angew. Chem. Int. Ed.* **2025**, *137*, e202504898.

23. Wichmann, L.; Aboobacker, A.; Heuvel, S.; Pfeiffer, F.; Hinz, R.-T.; Glorius, F.; Cekic-Laskovic, I.; Diddens, D.; Winter, M.; Brunklaus, G. Design of Fluorine-Free Weakly

Coordinating Electrolyte Solvents with Enhanced Oxidative Stability. *Angew. Chem. Int. Ed.* **2025**, *64*, e202506826.

24. Zhou, Z.; Yang, S.; Wu, J.; Cao, Y.; Huang, Y.; Yin, X.; Wang, X.; Zhao, Y.; Duan, J.; Luo, Z.; others Heteroatom-Tuned Weakly Solvating Electrolyte with Good Wettability Enriching Solid Electrolyte Interphase Chemistry toward Stable Lithium Metal Batteries. *J. Energy Chem.* **2025**,

25. Chemspace. `https://chem-space.com`, Accessed: 2026-04-27.

26. Molport. `https://www.molport.com`, Accessed: 2026-04-27.

27. Jin, B.; Lai, T.; Manthiram, A. Locally Confined Polysulfide-Reactive Electrolytes for Shuttle-Free Sodium–Sulfur Batteries. *J. Am. Chem. Soc.* **2025**, *147*, 26414–26424.

28. Ganguli, B.; Klevay, A.; Bhargav, A.; Long, J. Electrolyte Systems Including Components for Improving Performance of Lithium-based Secondary Batteries. WO Patent WO2025188344 A1, 2025.

29. Wu, K.; Li, X.; Guo, B.; Zhang, X.; Chen, S.; Yao, S.; Liu, B.; Zhou, Q.; Lan, J. Electrolyte, Secondary Battery and Electric Device. CN Patent CN119275344 A, 2025.

30. Zang, X.; Shen, D.; Li, Z.; Zheng, Z. Nonaqueous Electrolyte and Secondary Battery. CN Patent CN109119598 A, 2019.

31. Yamada, I.; Kubota, T. Nonaqueous Electrolyte Battery. US Patent US20110200885 A1, 2011.
